# Metabiotics Signature through Genome Sequencing and In Vitro Inhibitory Assessment of a Novel *Lactococcus lactis* Strain UTNCys6-1 Isolated from Amazonian Camu-Camu Fruits

**DOI:** 10.3390/ijms24076127

**Published:** 2023-03-24

**Authors:** Gabriela N. Tenea

**Affiliations:** Biofood and Nutraceutics Research and Development Group, Faculty of Engineering in Agricultural and Environmental Sciences, Universidad Técnica del Norte, Av. 17 de Julio s-21, Barrio El Olivo, Ibarra 100150, Ecuador; gntenea@utn.edu.ec or gtenea@hotmail.com

**Keywords:** metabiotics, *Lactococcus lactis*, pangenome, antimicrobials, exopolysaccharides, SemiSWEET sucrose transporter

## Abstract

Metabiotics are the structural components of probiotic bacteria, functional metabolites, and/or signaling molecules with numerous beneficial properties. A novel *Lactococcus lactis* strain, UTNCys6-1, was isolated from wild Amazonian camu-camu fruits (*Myrciaria dubia*), and various functional metabolites with antibacterial capacity were found. The genome size is 2,226,248 base pairs, and it contains 2248 genes, 2191 protein-coding genes (CDSs), 50 tRNAs, 6 rRNAs, 1 16S rRNA, 1 23S rRNA, and 1 tmRNA. The average GC content is 34.88%. In total, 2148 proteins have been mapped to the EggNOG database. The specific annotation consisted of four incomplete prophage regions, one CRISPR-Cas array, six genomic islands (GIs), four insertion sequences (ISs), and four regions of interest (AOI regions) spanning three classes of bacteriocins (enterolysin_A, nisin_Z, and sactipeptides). Based on pangenome analysis, there were 6932 gene clusters, of which 751 (core genes) were commonly observed within the 11 lactococcal strains. Among them, 3883 were sample-specific genes (cloud genes) and 2298 were shell genes, indicating high genetic diversity. A sucrose transporter of the SemiSWEET family (PTS system: phosphoenolpyruvate-dependent transport system) was detected in the genome of UTNCys6-1 but not the other 11 lactococcal strains. In addition, the metabolic profile, antimicrobial susceptibility, and inhibitory activity of both protein–peptide extract (PPE) and exopolysaccharides (EPSs) against several foodborne pathogens were assessed in vitro. Furthermore, UTNCys6-1 was predicted to be a non-human pathogen that was unable to tolerate all tested antibiotics except gentamicin; metabolized several substrates; and lacks virulence factors (VFs), genes related to the production of biogenic amines, and acquired antibiotic resistance genes (ARGs). Overall, this study highlighted the potential of this strain for producing bioactive metabolites (PPE and EPSs) for agri-food and pharmaceutical industry use.

## 1. Introduction

Functional metabolites secreted by probiotics are currently called metabiotics, referring to “structural components of probiotic microorganisms and/or formulations and/or signaling molecules with a determined chemical composition that have the potential to optimize host-specific physiological functions and regulate metabolic and/or behavior reactions connected with the activity of host indigenous microbiota” [1]. Organic acids, glycoproteins, bacteriocins, exopolysaccharides, polyphosphates, acetate, propionate, and butyrate constitute the major well-defined structural substances that constitute metabiotics [2]. Various probiotic strains are a source for thousands of metabolites that contribute to genome stability, cell-to-cell communication, and epigenetic regulation, making them attractive candidates for incorporation into the human diet [3]. In vivo production of these molecules relates to the producer microorganism and/or the prebiotic utilization capacity [4]. The first generation of human-derived probiotics was intended for the correction of microecological disorders [1], and its performance in metabiotic production in a food matrix is to be considered. Nonetheless, the potential for new probiotic strains originating from unconventional sources may be an attractive option for gut bacteria, as they have the potential to exhibit specific metabolic traits (enzymes) and health properties, beyond their technological functionality [3].

Due to its usage as a starter in cheese manufacturing, one of the most commercially significant LAB strains is *Lactococcus lactis*, which is also a promising probiotic candidate with numerous uses in the animal feed, pharmaceutical, therapeutic, and food industries [5]. Most characterized *L. lactis* strains have been isolated from the human and animal gut and have been listed by the U.S. Food and Drug Administration (FDA) with Generally Regarded as Safe (GRAS) status. Nonetheless, an endless number of novel *Lactococcus* species isolated from plant materials showed various technological benefits, as they generate organic acids and aromatic compounds that can improve the performance of the tested products [6].

The Amazon region constitutes a very diverse geographical space. The productive potential and microbiota associated with plants and fruits from this region remain unknown. We conducted a prospecting of many wild fruits from Ecuador’s Amazon rainforest to select native LABs for biotechnological usage [7]. Only a few lactococcal isolates have been found and characterized in vitro [8]. The microbial population associated with these niches, differing among samples, as a plant matrix, might carry a particular microbiota in a specific geographical region and at a specific time point [9]. Considering the benefits of *L. lactis* strains as starter cultures or additives that prevent spoilage or spread of pathogens in food, together with their unique metabolic profile and health-promoting properties, we conducted a whole-genome sequence analysis of a novel UTNCys6-1 isolate originated from wild Amazonian camu-camu fruits (*Myrciaria dubia* H.B.K Mc Vaugh). Camu-camu, locally known as “manzana silvestre”, is a wild bush fruit with the highest vitamin C content that contributes to improving the quality of life due to its positive effect on health [10]. The isolate was classified taxonomically, and the phylogeny with other related strains was assessed. Gene mapping was performed to detect the potential involvement of the predicted genes in different biological pathways and to identify genes encoding for probiotic and metabiotic traits. Moreover, the presence of CRISPR sequences, mobile genetic elements, biogenic amine production, antibiotic resistance genes, and virulence factors was assessed using various genome mining tools. In addition, pangenome analysis was performed to identify core, accessory, and unique proteins and to search for the presence or absence of genes among 11 lactococcal species. In addition, the antibiotic sensitivity and metabolic profile along with the capacity to generate natural biopolymers such as EPSs was evaluated in vitro. In addition, the effect of peptide–protein extract (PPE) and EPSs (obtained from both solid and liquid sugar-containing media) was tested to determine their inhibitory capacity on specific foodborne pathogens. These findings are important for supplying information on genomic diversity, molecular evolution, and safety, as well as the capacity to produce relevant functional metabolites for additional biotechnological applications.

## 2. Results and Discussion

### 2.1. Species Identification and Phylogenetic Relationship

**Figure 1:**
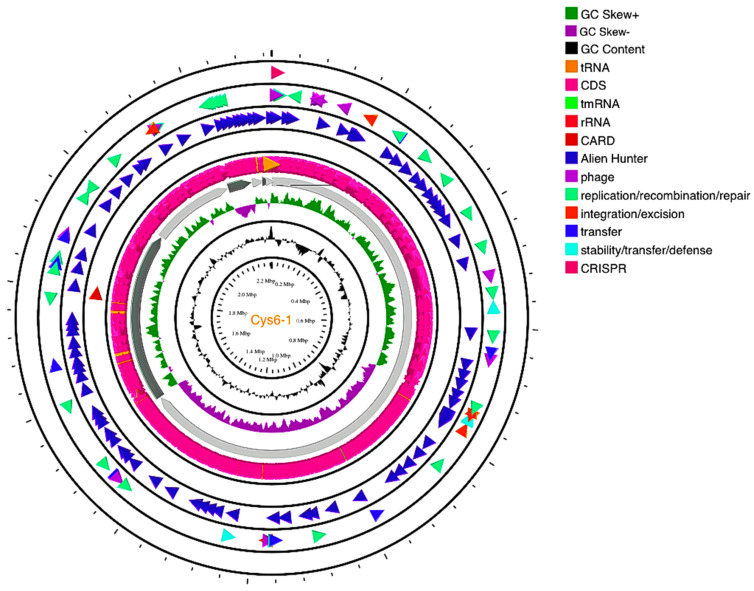
Circular maps of the UTNCys6-1 predicted with Proksee viewer (https://proksee.ca, accessed on 6 March 2023). The contents are arranged in feature rings (starting with the outermost ring): ring 1: CRISPR elements; ring 2: mobile genetic element (MGE) annotation with Mobile OG DB marking *hsdR* gene involved in stability/transfer/defense; ring 3: MGE annotation with Alien Hunter predicting horizontal genetic transfer (HGT) events (blue color arrows); ring 4 shows the UTNCys6-1 protein-coding genes (CDSs) with Prokka annotation (magenta color); tRNA, rRNA, and tmRNA are marked; ring 5 displays the GC content plot (black); ring 6 displays G/C skew information in the + strand (green color) and − strand (purple color).

**Figure 2:**
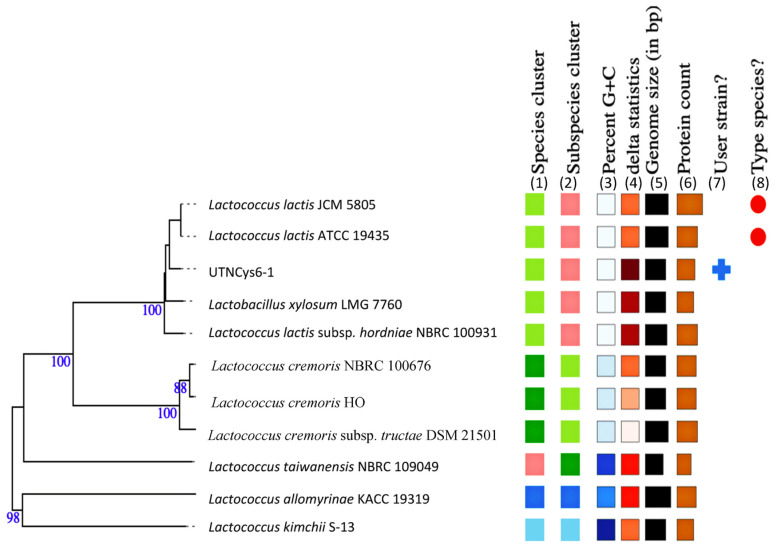
Phylogenetic tree constructed on TYGS (https://tygs.dsmz.de/ accessed on 6 March 2023). Branch lengths are scaled in terms of GBDP (genome BLAST distance phylogeny method) distance; numbers above branches are GBDP pseudo-bootstrap support values from 100 replications. Leaf labels with different colors indicate the following: (1) species cluster; (2) subspecies clusters; (3) genomic G + C content (min 34.82–39.37); (4) δ values (min 0.061–max 0.17); (5) overall genome sequence length (1,950,384–2,758,410 bp); (6) number of proteins (1847–3118). (7) Target strain is marked. (8) Type species are marked.

A total contig of 2,331,682 bp with an estimated genome size of 2,226,248 bp was generated by sequencing analysis. A summary of the genome assembly data is shown in Table S1. To determine which species each framework showed an affinity for, BLAST analysis was used [11]. The proportion based on the genus level as the result of the best hit for the entire contig was 100% matching *Lactococcus.* The closest genome according to the ANI results was *L. lactis* D53 (GCF_012689205.1) with 99.25% nucleotide identity and 93.71% alignment coverage, followed by *L. lactis*_1001095IJ_161003_G11 (GCF_015551225.1) with 99.11% nucleotide identity and 89.27% alignment coverage and *L. lactis* LB6 (GCF_016649155.1) with 99.01% nucleotide identity and 89.83% alignment coverage, ranked according to the highest nucleotide identity. The hierarchical clustering of data in two dimensions was represented with dendrograms plotted with a simple linkage of ANI percentage identity and ANI alignment coverage (Figure S1). Based on the results, the UTNCys6-1 isolate was assigned as *L. lactis*. The genome map is shown in Figure 1. The closest strain genome type was determined by comparing the UTNCys6-1 genome to all accessible strain genomes in the TYGS database using the MASH algorithm [12]. Strains with the shortest MASH distance were selected automatically. Thus, the precise distance was calculated using the Genome BLAST distance phylogeny (GBDP) approach under the “coverage” algorithm and distance formula [13]. According to GBDP, the 12 most similar strain genomes were selected automatically from the database. The results of the whole-genome analysis placed the UTNCys6-1 strain on the same branch as *L. lactis* subsp. *hordniae* NBRC100931 and *L. lactis* JCM5805 (Figure 2). The UTNCys6-1 genome assembly data were deposited in the NCBI (Sequence Read Archive) database: BioProject PRIJNA847762 (https://www.ncbi.nlm.nih.gov/bioproject/?term=847762 and BioSample SAMN28955494 accessed on 10 June 2022).

### 2.2. Gene Prediction and Functional Annotation of Enzymes Involved in Different Metabolic Pathways

**Figure 3:**
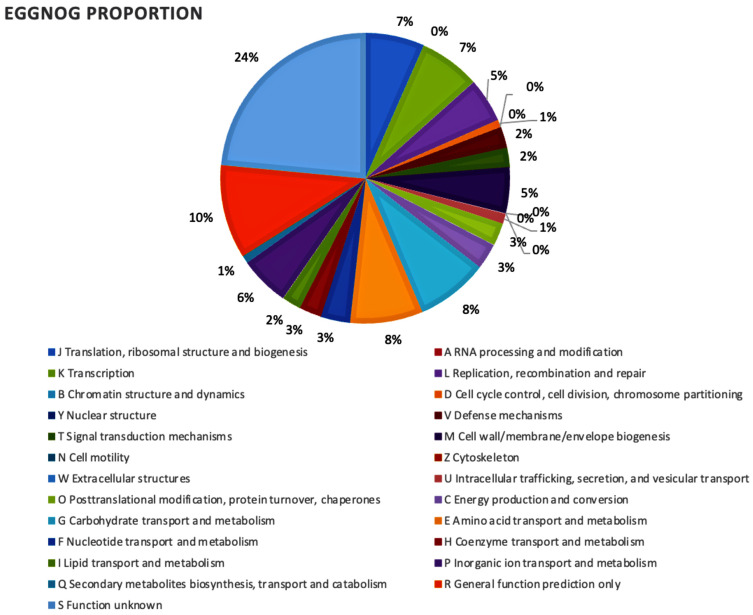
EggNOG category distribution of functional annotation result.

The genome contains 2191 CDSs, 50 tRNAs, 6 rRNAs, 1 16S rRNA, 1 23S rRNA, and 1 tmRNA. Among the total proteins, there were 2148 matched EggNOG DB proteins (2123 Single EggNOG and 25 Multi EggNOG proteins) and 43 proteins with no hit. Figure 3 depicts the EggNOG category distribution. Genomic analysis revealed that the UTNCys6-1 strain harbors several functional genes conferring the capacity to survive in stressful conditions, such as two hypothetical proteins such as sortase (the surface protein anchoring transpeptidase), the LPXTG motif, and exopolysaccharide phosphotransferase CpsY; some hypothetical proteins involved in the metabolism of carbohydrates and glycerol; and functional genes for the biosynthesis of vitamins and amino acids. The *sodA* gene coding for superoxide dismutase, known for its effect on radical scavenging in cells [14], has been annotated in the UTNCys6-1 genome. In addition, from genome annotation analysis, ten genes encoding the biosynthesis of riboflavin proteins (RibD, RibE, RibBA) and 6,7-dimethyl-8-ribityllumazine synthase (RibH), which catalyze the formation of 6,7-dimethyl-8-ribityllumazine as the penultimate step in the biosynthesis of riboflavin RibD, were detected. Early genome analysis of *L. lactis* IL1403 indicated the presence of 20 genes responsible for the synthesis of various amino acids and at least four cofactors (folic acid, menaquinone, riboflavin, and thioredoxin) [15].

Genome analysis pointed out that UTNCys6-1 harbored the *men* operon (*menABCDEFGH*), which encodes proteins necessary for the conversion of demethylmenaquinone (DMKH2) to menaquinone (MKH2) and catalyzes the conversion of acetate into acetyl-CoA (AcCoA), a substantial intermediate at the junction of anabolic and catabolic pathways. In addition, two genes (*hemH* and *hemN*) annotated with EggNOG are required for the oxidation of coproporphyrinogen III and attachment of iron to heme, suggesting the existence of aerobic respiration. Early proteome analysis indicated that the metabolism of *L. lactis* gradually switches from fermentation to respiration during development when oxygen and heme are provided [16]. Considering the origin of this strain, a fruit matrix, it is interesting to explore its ability to form fermentation products other than lactate; this activity depends on enzymes that act on the key metabolite as pyruvate. Consequently, several genes encoding enzymes such as pyruvate dehydrogenase E1 component (*pdhABCD*), α-acetolactate synthase (*als*), pyruvate formate-lyase 1-activating enzyme (*plfA*), pyruvate-formate acetyltransferase (*pflB)*, and l-lactate dehydrogenase (*ldhA* and *ldhB*) have been annotated in the UTNCys6-1 genome. In addition, a gene encoding for pyruvate oxidase, *poxL*, was annotated with EggNOG. The PTS system is important from an industrial standpoint for increasing the production of specific metabolites through metabolic engineering, as well as for understanding the molecular basis of the variation between various types of fermentation [16].

### 2.3. Prediction of CRISPR Elements, Prophages, ARGs, VFs, GIs, ISs and Pathogenicity

A sequence with CRISPR located in contig 1 (beginning at 9861 and ending at 9950) and encompassing a short spacer sequence spanning between degenerate repeats (consensus DRs) with 95.83% conserved repeats and 100% spacer conservation was found in the UTNCys6-1 genome (Figure 1). No Cas elements were detected. Previous studies have shown that *L. lactis* lacks the CRISPR-Cas system, and protection systems may rely on other mechanisms, such as blocking phage adsorption, immunity, restriction modification, and abortive infection mechanisms [17]. A few *L. lactis* strains harbor a CRISPR-Cas type III-A system with a conjugative plasmid that provides resistance to virulent phages from the core of the *Siphoviridae* family [18]. Further, the results indicated that UTNCys6-1 isolate is not a human pathogen (0.20 likelihood), as evidenced by the absence of any pathogen family. Complementary hemolytic activity on blood agar media indicated that UTNCys6-1 is not a pathogenic strain, as no formation of hemolytic zones was observed. There was a total of four incomplete prophages within the contigs 1, 2, and 3 of the UTNCys6-1 genome spanning a total of 68 proteins (Table S2). The most common phages were PHAGE_Lactoc_bIL286_NC_002667(3), PHAGE_Lactoc_TP901_1_NC_002747(2), PHAGE_Lactoc_bIL310_NC_002669(8) and PHAGE_Paenib_Yerffej_NC_048714(1). Research into phage–*L. lactis* interactions has revealed an extensive repertoire of diverse phage defense mechanisms. In this study, based on the EggNOG analysis, two hypothetical proteins of the “K category” antirepressor prophage (COG3617), transcriptionally related, were annotated in the UTNCys6-1 genome with a hit specific for the BRO-N family. Members of this family integrate the N-terminus of BRO baculovirus and ALI core proteins with unknown functions. It is proposed that BRO-A and BRO-C are DNA-binding proteins that affect the replication and/or transcription of host DNA [19]. The homology of the BLASTP protein sequence and protein alignment showed 100% identity with the phage antirepressor protein from *L. lactis* subsp. *lactis* (KST84049.1). The genetic basis for many of these naturally occurring systems has been identified, which helps us understand how phage–host interactions have evolved and makes it possible to use them in strain development to increase phage resistance [20].

Based on PlasmidFinder, no plasmids were detected in the UTNCys6-1 genome. Additionally, a total of six islands (GIs) with a length of 126933 bp were predicted with IslandViewer using as reference the *L. lactis* subsp. *lactis* IL1403 genome. Most of them encode several hypothetical proteins and proteins involved in the defense mechanism, such as lantibiotic nisin_Z, nisin biosynthesis protein (NisB, NisC), nisin immunity protein (NisI), and transport ATP-binding protein LagD, nisin leader peptide-processing serine protease (NisP), and putative ABC transporter ATP-binding protein (YxlF), which were detected within the genome of UTNCys6-1 (Table S3). These bacteriocins might support the strain’s overall defense and niche adaption. Additionally, several site-specific tyrosine recombinases Xer (XerD 1, XerD 2, XerD 3, XerS1, XerS2), which have been identified in *E. coli* K12 and catalyze the cleavage and ligation of recombinant DNA molecules, were discovered in the GI area [21]. No pathogen-associated genes, VFs, or ARGs were discovered in GIs.

Moreover, a total of four ISs, grouped into two families, were registered with the ISfinder web tool. The UTNCys6-1 genome harbors two distinct transposases, ISNCY and ISL3 (3), according to ISsaga annotation. The EggNOG annotation indicates the presence of the IS1595 nuclear family ISCac2 transposase with dihydrofolate reductase activity (COG0262). A larger number of ISs (43) organized into six families with non-random chromosomal distribution were previously reported in the *L. lactis* IL1403 strain [15]. Additionally, UTNCys6-1 is free of VFs. The UTNCys6-1 genome was predicted to include an ATP-binding cassette (ABC) antibiotic efflux pump using CARD analysis (Figure S2). BLAST analysis of the gene *lmrA* showed sequence similarity with different *L. lactis* strains. The sequence showed a specific hit to an ABC-type multidrug transport system (COG1132), a component of defense mechanisms. EggNOG annotation and pangenome analysis confirmed the presence of such genes across the *Lactococcus* species (11 strains considered in this study). In addition, a tetracycline resistance gene was predicted from CARD analysis in the genome of the UTNCys6-1 strain. Nonetheless, the resistance was not confirmed by in vitro antibiotic sensitivity (Table S4). Early studies identified several *Lactococcus lactis* strains isolated from dairy and animal products showing intrinsic resistance to tetracycline [22]. Nonetheless, the European Food Safety Authority (EFSA) defines “intrinsic resistance” as a characteristic of antimicrobial resistance that is common within a single species but does not present a safety issue [23].

Consistent with the EggNOG genome annotation (COG0188) and CARD prediction, a gene (*parC*) involved in fluoroquinolone resistance was detected in the genome of the UTNCys6-1 strain. BLASTP analysis showed that this protein has a 100% sequence identity with DNA topoisomerase IV subunit A of *L. lactis* (MBS7066896.1). DNA topoisomerases are critical enzymes that regulate conformational changes in DNA topology by catalyzing the concerted breakage and rejoining of DNA strands throughout normal cell growth [24]. Nonetheless, its level of expression is not yet known. The ResFinder examination of the genome revealed any acquired antibiotic resistance genes, demonstrating the stability of the strain genome. Additionally, the genome annotation analysis revealed no genes involved in the production of biogenic amines (such as arginase, arginine decarboxylase, histidine decarboxylase, lysine decarboxylase, ornithine/lysine decarboxylase, spermidine synthase, ornithine decarboxylase, tyrosine decarboxylase, and tryptophan decarboxylase). These results suggested that the target UTNCys6-1 is a safe strain; additional in vivo testing is required to confirm this statement.

### 2.4. Pangenome Comparison Analysis

**Figure 4:**
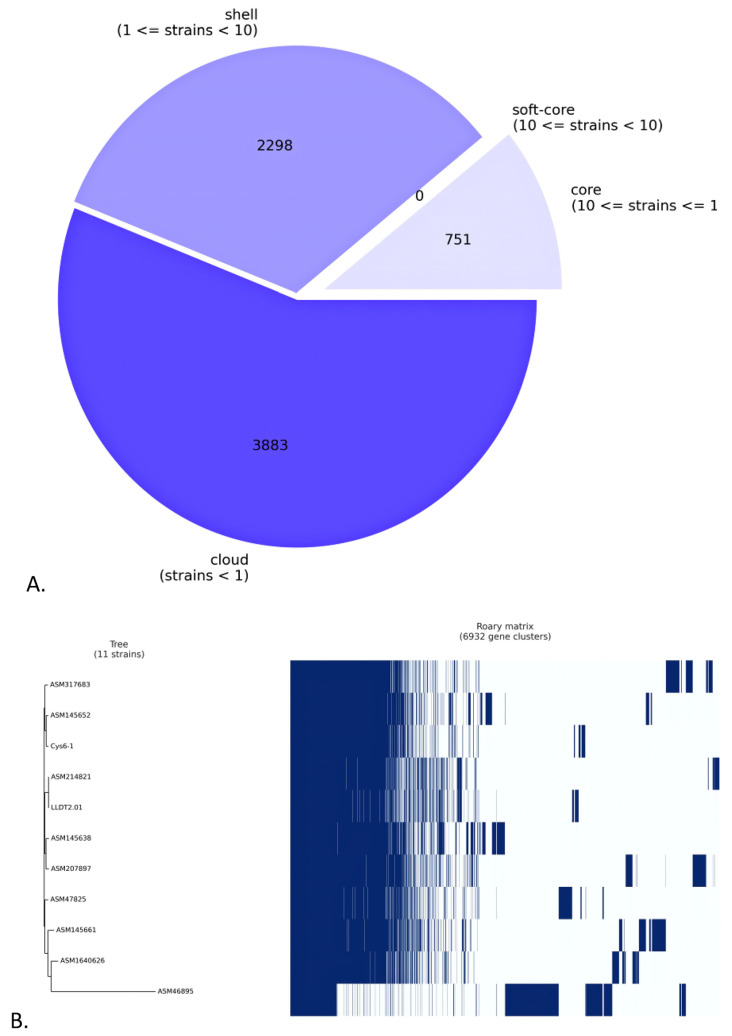
(**A**) A pie chart depicting the number of genes in the UTNCys6-1 strain’s core, soft core, shell, or cloud. (**B**) A comparison of the genetic content of the 11 strains considered. The matrix depicts the typical genes of each strain as well as those that are conserved among them. The genome assembly codes are described in Table S6.

A comparative pangenome analysis of the UTNCys6-1 genome and the ten *L. lactis* selected genomes was performed. Based on this study, we found 6932 sets of genes, of which 751 (core genes) were commonly observed in the 11 phyla. Among them, 3883 genes were sample-specific genes (cloud genes), 2298 were shell genes, and any gene within the soft core was detected, indicating its high genetic diversity (Figure 4A). The matrix of gene content comparison of the 11 strains is shown in Figure 4B. Furthermore, by analyzing the presence and absence of genes among the 11 strains, several species-specific protein-coding genes were discovered. A *ltrA* gene was detected in the genome of UTNCys6-1 but not the other 10 *Lactococcus* strains. EggNOG annotation confirmed the presence of LtrA protein, a multifunctional protein that promotes group II intron splicing and mobility by acting both on RNA and DNA [25]. This gene was predicted within the genomic islands. In addition, *lacF_1*, *gmuB*, and *licC* genes encoding for PTS system lactose-specific EIIA component, PTS system oligo-beta-mannoside-specific EIIB component, and lichenan permease IIC component, respectively, were detected in the UTNCys6-1 genome only. The enzyme II LacEF PTS system is involved in lactose transport and was previously detected in *Lactobacillus casei* [26]. The enzyme system GmuABC PTS II is involved in the transport of oligo-glucomannans such as cellobiose and mannobiose and was detected in the genome of *Bacillus subtilis* [27]. The enzyme LicC is involved in lichenan transport and is part of the PTS transporter system previously identified in the *B. subtilis* genome [28]. The BLASTP assay showed 99.77% identity with the EIIC subunit of the *L. lactis* PTS transporter (WP_235585325.1). The EggNOG and pangenome study revealed a *dapH_1* gene encoding 2,3,4,5-tetrahydropyridine-2,6-dicarboxylate-N-acetyltransferase in the UTNCys6-1 genome but not in the other 10 genomes. This enzyme (COG0584) was assigned “Category C” for energy production and conversion using the EggNOG mapper and was previously recognized in the genome of *Staphylococcus aureus* [29].

A sugar transporter of the SemiSWEET family, harboring a PQ motif (Carbohydrate transport and metabolism) (COG4095), was detected in the UTNCys6-1 genome but not in the other 10 genomes. BLASTP analysis against the protein database showed a 43.71% sequence identity with the choline-binding protein A sequence from *Ligilactobacillus salivarius* (locus 5GT1_A). SemiSWEET sucrose transporters are found in both eukaryotes and prokaryotes; nonetheless, their functionality is not well understood [30]. Members of this family are known as membrane-bound proteins comprising a pair of repeats, each spanning two transmembrane helices connected by a loop. Based on EggNOG annotation, this transporter was located downstream of genes involved in carbohydrate transport and sugar metabolism, such as the *lacF_1* gene of PTS system lactose-specific EIIA component, with specific hits on the lactose- and cellobiose-specific IIA subunit multiprotein system (COG1447). Likewise, pangenome analysis reveals that the *lacF_1* gene is unique to the UTNCys6-1 genome. The IIA component of the lactose/cellobiose transporter PTS from *L. lactis* (WP 058217751.1) exhibited 100% similarity when compared using BLASTP against the protein database. The lactose/cellobiose-specific family is one of the four enzymes of the structurally and functionally diverse group IIA PTS system [31]. However, the PTS system helps bacteria to habituate certain niches [32]. Consistent with the EggNOG annotation, the UTNCys6-1 genome harbors a *cpsY* gene encoding the exopolysaccharide phosphotransferase CpsY and a hypothetical protein involved in exopolysaccharide biosynthesis. Among the genomes, CpsY was also detected in *L. lactis* ATCC19435. BLASTP results showed 100% sequence identity with *L. lactis* glycosyltransferase (locus WP_226319360.1). The UTNCys6-1 query sequence revealed specific hits on the CR2 stealth protein (conserved region 2), a member of some highly conserved regions of capsular polysaccharide phosphotransferases that are involved in the defense mechanisms [33]. The pangenome analysis revealed significant genomic heterogeneity among the studied strains, which may explain the strain’s habituation to various micro-niches and their biotechnological potential.

### 2.5. Metabolite Gene Cluster (MGC) Prediction

From the UTNCys6-1 FASTA query genome sequence analysis, five MGC regions were identified within the contigs 1, 3, and 4, including region 3.1 (location: 85,098–111,226 nucleotides), of arginine2_Hcarbonate (Table 1). The ClusterBlast region output indicated that this region showed 100% similarity to *L. lactis* strain AM11-49, while KnownClusterBlast indicated 100% similarity to arginine-2-hydrogen carbonate of *Pseudomonas aeruginosa*. Likewise, contig 1 contains three regions of gene clusters, type GR_AA metabolism, type TPP_fatty_acids, and type TPP_AA_metabolism with unknown function. In addition, contig 4 has a set of other_HGD_unassigned zone-type genes with unknown functions that do not fit into a GutSMASH-assigned category (Table 1). Early studies revealed that arginine metabolism in sucrose-depleted *L. lactis* enhanced cell viability and survival, as well as cheese flavor synthesis [34]. Additionally, *L. lactis* ATCC11474 genome contains the Arginine2 Hcarbonate gene cluster with 100% identity to *L. lactis* strain AM11-49.

Furthermore, by using contigs as input in the antiSMASH web tool, five regions, namely beta-lactone (RiPP-like), lanthipeptide-class-I (RiPP-like), T3PKS, and two RaS-RiPP (RaS radical S-adenosyl-l-methonine-RiPP) regions were predicted (Table 1). Among the beta-lactones, various compounds such as coagulin, sublancin, and duramycin were predicted in the UTNCys6-1 genome, but any known ClusterBlast output was detected (Table S5A). Furthermore, in Class 1 lanthipeptides, the most similar gene cluster was nisin_A, which shared 100% identity with *L. lactis* strain 21 and *L. lactis* subsp. *lactis* strain F44. In the RaS-RiPP category, various compounds such as streptide, subtilin, and ericin have been predicted within the UTNCys6-1 genome. RaS-RiPP operons usually encode a precursor peptide, a RaS enzyme, in some cases an RRE protein (RiPP recognition element), and additional genes for modification and transport [35]. Within the *L. lactis* ATCC11474 genome, a total of seven similar regions were detected, with two additional RaS-RiPP categories compared to UTNCys6-1 (Table S5A,B). Further research is needed to assess the impact of these regions on the strain’s technological potential.

### 2.6. BGC Organization Predicted from Genome Study

**Figure 5:**
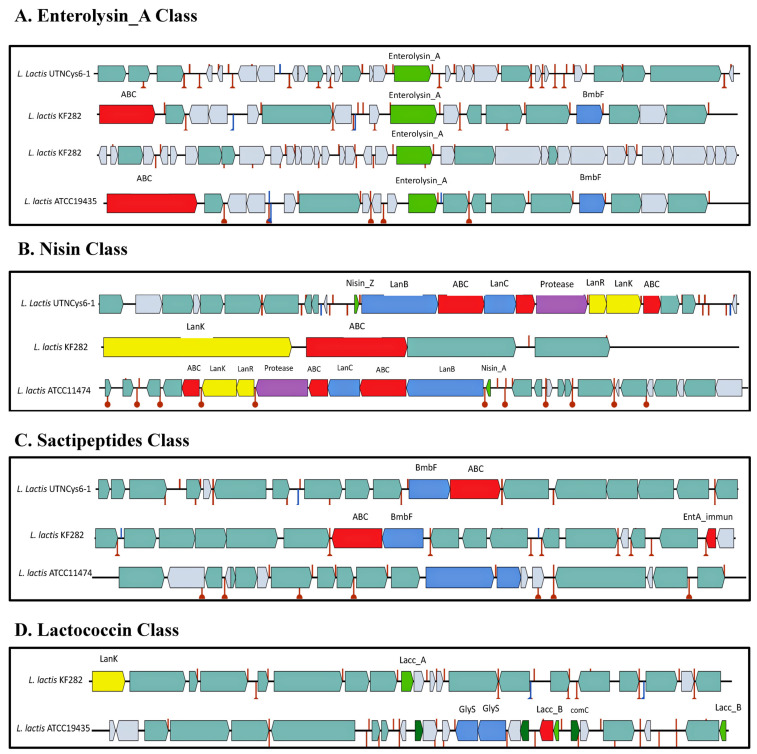
Bacteriocin gene cluster organization comparison between *L. lactis* UTNCys6-1 and the references *L. lactis* ATCC19435, *L. lactis* KF282, and *L. lactis* subsp. *lactis* ATCC11474 strains. (**A**) Enterolysin_A class; (**B**) nisin_Z class; (**C**) sactipeptide class; (**D**) lactococcin class. Genes with determined function: enterolysin_A: ABC: bacteriocin ABC transporter; BmbF: uncharacterized protein MJ0907 (*Methanocaldococcus jannaschii* ATCC 43067); Nisin_Z: leaderLanBC (gallidermin); LanB: nisin biosynthesis protein NisB (*L. lactis* subsp. *lactis* 1360); ABC: nisin transport ATP-binding protein nisT; LanC: nisin biosynthesis protein nisC; orf00024: nisin immunity protein; protease: nisin leader peptide-processing serine protease NisP (*L. lactis* subsp. *lactis* 1360); LanR: nisin biosynthesis regulatory protein nisR; LanK: nisin biosynthesis sensor protein nisK; ABC: NisF; orf00032: NisE; orf00033: NisG; EntA_immun: bacteriocin immunity protein; Gly (orf00023): plantaricin biosynthesis protein PlnO; Gly (orf00024): lipopolysaccharide core biosynthesis glycosyltransferase WaaE. Legend: red blocks: immunity and transport; green blocks and green arrows: core peptide; pink blocks: transport and leader cleavage; blue blocks: peptide modifications; yellow blocks: regulation; grey blocks: no function determined; maroon line with circle ends are the predicted terminators.

Four bacteriocin clusters (areas of interest (AOIs)) were annotated within the UTNCys6-1 genome as follows: contig 1.3 (AOI_01) (started at 1161027, ended at 1181561) and contig 1.3 (AOI_02) (started at 71843, ended at 92287) of the Enterolysin_A class, contig 1.3 (AOI_03) (started at 126719, end at 151879) of the Nisin_Z class, and contig 2.4 (AOI_04) (started at 32831, ended at 52831) of the sactipeptide class (ribosomally synthesized peptides) (Figure 5). The new isolate UTNCys6-1 harbors two genes encoding enterolysin_A, a cell-wall-degrading bacteriocin that was previously detected in *Enterococcus*, *Lactococcus*, and *Streptococcus* [36]. The BLASTP results indicated that enterolysin_A from AOI_01 corresponds to an M23 family of metallopeptidases, which induces the lysis of cell wall peptidoglycans, such as the endopeptidase l-Ala-d-Glu from *Bacillus subtilis* [37]. The enterolysin_A from AOI_02 showed specific hits with lysozyme, endolysin, and autolysin enzymes, and another specific hit with M23 peptidase. Similarly, one enterolysin_A gene cluster was detected in contig 15 of *L. lactis* ATCC19435 strain, and two enterolysin_A gene clusters were detected in contigs 9 and 13 of *L. lactis* subsp. *lactis* strain KF282 (Figure 5A). No enterolysin_A cluster was detected within the *L. lactis* ATCC11474 genome. Enterolysin-A is a high-molecular-weight protein [38] which in combination with another peptide/protein and molecules secreted in the precipitated cell-free supernatant might enhance the inhibitory activity. This statement should be further investigated.

AOIs within contig 1.3 consist of a lantibiotic, nisin_Z (LanBC leader) located downstream of nisin biosynthetic proteins (NisB, NisC, NisR, NisK, NisF, NisE, NisG), nisin transport ATP-binding protein (NisT) (orf00020), immunity protein nisin (orf00024), and a nisin leader peptide processing serine protease (NisP) (Figure 5B). The BLASTP analysis indicated 100% identity with gallidermin/nisin, a member of the lantibiotic family found in *Lactococcus* (WP_015425978.1). The query sequence showed a conserved domain similar to nisin_A. Nisin Z differs from the typical nisin A peptide by only one amino acid, resulting in greater solubility at physiological pH, making it a more suitable candidate for biomedical applications [39]. Likewise, the nisin_Z class was found in *L. lactis* KF282 but not in *L. lactis* ATCC19435 or *L. lactis* ATCC11474 (Figure 5B). In addition, *L. lactis* ATCC11474 contains the nisin A cluster gene which includes 11 genes (*nisABTCIPRKFEG*) encoding functions such as synthesis of the nisin precursor (nisA), regulation of nisin biosynthesis (nisRK), the processing and translocation of nisin (nisBCTP), and immunity (nisIFEG). Pangenome analysis indicated the presence of *nisZ* in 3 of 11 lactococcal strains; thus, only some of the *L. lactis* strains are nisin producers. Almost similar gene organization of the sactipeptide class was predicted for the UTNCys6-1 and KF282 strains, but this organization was divergent from that of *L. lactis* ATCC11474 (Figure 5C). This class contains an ABC transporter ATP-binding protein containing both ATPase and permease components of an ABC-type multidrug transport system involved in a defense mechanism showing 100% identity with various *Lactococcus* species. In addition, it contains a BmbF gene encoding for an uncharacterized protein from *Methanocaldococcus jannaschii* ATCC43067. The BLASTP study indicated that this protein has specific hits to the SAM radical superfamily of proteins that catalyze various reactions, including unusual methylation, isomerization, sulfur insertion, ring formation, anaerobic oxidation, and the formation of protein radicals [19]. In addition, KF282 harbors a bacteriocin immunity-encoding gene (EntA_immun). Bacteriocins of the sactipeptide class were not predicted in the *L. lactis* ATCC19435 genome, whereas both reference KF282 and ATCC19435 strains possess lactococcal class bacteriocins (Figure 5D). The lack of genes from the lactococcal class in UTNCys6-1 and *L. lactis* ATCC11474 suggests that the antibacterial activity is a result of different compounds that are strain-specific and does not depend on the presence or absence of a particular bacteriocin class. This is consistent agrees with our early observations, which indicated that the *L. lactis* strain UTNGt28 harboring genes encoding lactococin A, lactococin M, and lacticin showed high antimicrobial activity against food pathogens, despite lacking nisin-encoding genes [7].

### 2.7. In Vitro Characteristics

#### 2.7.1. Metabolic Profile and Antibiotic Susceptibility

The sugar assimilation/acid formation test performed on the BBL Crystal revealed positive results for trehalose, lactose, sugar, fructose, mannitol, glycerol, esculin, ferric citrate, proline, leucine, cellobiose, maltose, melibiose, sugar, and glycoside, whereas H2S production, arginine, and urease were negative (Figure S3). An *fbp* gene encoding class 3 fructose-1,6-bisphosphatase was annotated with EggNOG, supporting the positive metabolic results. Initial findings indicate that FBPase catalyzes the conversion of fructose-1,6-diphosphate to fructose-6-phosphate, which is a substantial step in the biosynthesis of sucrose nucleotides from fructose [40]. In addition, a gene encoding sucrose-6-phosphate hydrolase (*scrB*) and a putative fructokinase (*gmuE_1*) were annotated in the GI region of UTNCys6-1. According to the pangenome analysis, the *fbp* and *scrB* genes were found in 10 and 6, respectively, of the 11 studied strains’ genomes. The PTS system component GmuE_1 was exclusively found in the UTNCys6-1 genome. Furthermore, lactococcal strains lacking genes producing lactate dehydrogenase (LDH) were found to produce mannitol from glucose [41]. Additionally, lactose, the major carbohydrate in the dairy niche, was fermented by both strains. Several genes involved in lactose metabolism have been identified from the genome annotation, including lactose permease (*lac5*), which is involved in carbohydrate transport (symporter activity), and aldose 1-epimerase (*mro*), which converts alpha-aldose to beta-anomer and is active on d-glucose, l-arabinose, d-xylose, d-galactose, maltose, and lactose. In addition, the strain may be suitable to be tested in dairy niches due to the presence of beta-galactosidase (lacZ) [42]. The use of glycerol as a growth substrate can be helpful from a biotechnological standpoint, as glycerol by-products have been investigated as a raw material to develop lactic acid [43]. EggNOG results showed that the UTNCys6-1 genome contains genes for the xylose activator XylR (*xylA*, *xylB_1*, and *xylB_2*) as well as enzymes and proteins for xylose and xylulose consumption. The presence of these genes in the 11 strains was confirmed through a pangenome study. Likewise, certain genes associated with the utilization of d-ribose (rbsK/rbiA) and ribokinase (rbsK) were annotated within the UTNCys6-1 genome.

The susceptibility to certain common antibiotics was evaluated [44]. The results revealed that the UTNCys6-1 strain was resistant to gentamycin but susceptible to penicillin, ampicillin, amoxicillin, cefotaxime, tetracycline, and erythromycin (Table S4). The outcomes matched the reference antibiotic profile for *L. lactis* ATCC11474. According to earlier research, multidrug-resistant efflux pumps are the main cause of antibiotic resistance in LAB strains [45]. However, some *Lactococcus* strains isolated from a traditional fermented beverage (corn-based) have active efflux pumps, integrated with the chromosomally encoded ABC LmrA transporter (*lmrA* gene). As discussed above, an ATP-binding cassette (ABC) antibiotic efflux pump was predicted by CARD and annotated with EggNOG in the current investigation. Likewise, the pangenome analysis confirmed the presence of the *lmrA* gene among the 11 genomes tested. Similarly, a *lmrD* resistance gene was predicted in the *L. lactis* ATCC11474 strain (data do not show). In another study, the LmrA transporter in *L. lactis* was linked to innate resistance to 21 clinically relevant antibiotics, including aminoglycosides (kanamycin and gentamycin), lincosamines (clindamycin), macrolides (erythromycin), quinolones (ciprofloxacin), and tetracyclines [46]. Overall, considering that no acquired antibiotic resistance gene was found, and from these preliminary in vitro analyses, we concluded that the UTNCys6-1 strain is a safe strain; a complete set of phenotypic antibiotic resistances should be addressed before it is considered as probiotic strain.

#### 2.7.2. Inhibitory Activity against Foodborne Pathogens

**Figure 6:**
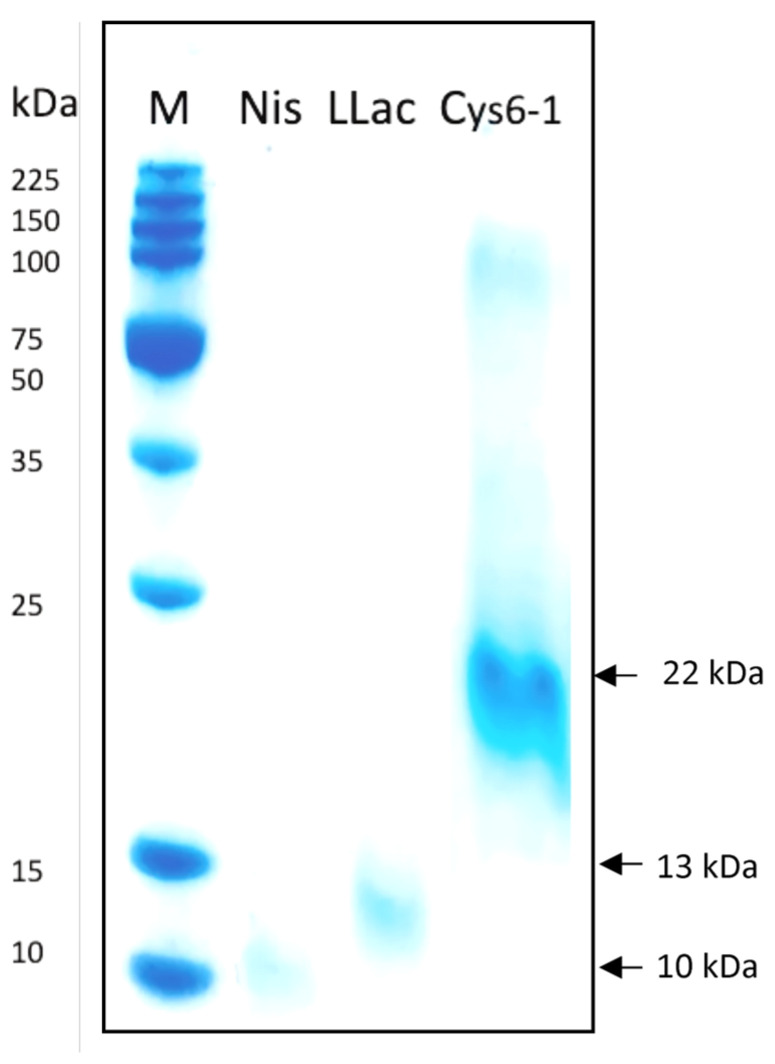
Estimation of UTNCys6-1 molecular weight. Legend: M: broad-range molecular marker (Promega); Nis: commercial nisin from *L. lactis*; L.Lac: PPE obtained from *L. lactis* subsp. *lactis* ATCC11474; Cys6-1: *L. lactis* UTNCys6-1.

The inhibitory activity of PPE, EPSS, and EPSQ was evaluated against several indicator strains (Table 2). Previous studies showed that several *Lactococcus* strains had a high inhibitory spectrum against Gram-positive bacteria, including *Listeria*, and this activity was linked to nisin secretion [47]. Using complementary analysis, the molecular weights of the PPEs produced by UTNCys6-1 and L.LAC were estimated at 22 kDa and 13 kDa according to Tricine-SDS-PAGE analysis (Figure 6). Under these experimental conditions, the size was larger than that of *Lactococcus* nisin-producer strains, suggesting that extra molecules could associate into a larger complex compound. An early study showed that *L. lactis* strain GLY32 isolated from boza produced a bacteriocin of 6.7 kDa [48]. Likewise, in this study, the molecular size of commercial nisin was about 10 kDa on SDS-PAGE. Previous reports indicated that nisin has a size of 3.5 kDa, and its inhibitory impact has been well demonstrated [49]. Commercial nisin has 2.5% pure nisin along with insoluble material which, in this analysis, influenced gel migration, resulting in a larger stained product. Nonetheless, nisin is still the only bacteriocin produced on an industrial scale and approved as a food additive [50]. In addition, it requires a chelator (EDTA) to have antibacterial effects on both Gram-negative and Gram-positive bacteria [47]. In a supplemental antimicrobial assay, molecular weight cutoff (MWCO) fractions of 3.0, 5.0 and 10 kDa of UTNCys6-1 and L.LAC were obtained from PPE filtration using membranes (Vivaspin 500, Sartorius, Göttingen, Germany) and were evaluated against *Listeria.* The 5.0 kDa fraction of UTNCys6-1 showed an inhibitory halo of 11.66 ± 0.58 mm, while L.LAC showed no activity. The 3.0 kDa fraction of L.LAC showed an inhibitory halo of 11.33 ± 0.49 mm, but UTNCys6-1 did not. For both strains, an identical inhibitory zone (10.58 ± 0.01 mm) matching the 10 kDa threshold fraction was recorded. Given that the inhibition zone of PPE without fractionation was higher, this implies that more than one active molecule, or a combination thereof, as well as other substances secreted in the extract, may control the total inhibitory ability (Table 2). To ascertain the molecular mechanism of action, molecule composition, and identification of these fractions, more investigations are necessary.

Various *Lactococcus* strains produce EPSs with unusual inhibitory capacity against pathogenic microorganisms [51]. EPSs from *L. lactis* strain F-mou (LT898177.1) isolated from Sahrawi camel milk in Algeria showed strong inhibitory activity against *S. aureus*, *P. aeruginosa*, *E. coli*, *L. monocytogenes*, *B. cereus*, *Proteus mirabilis*, *Acinetobacter baumannii*, *Enterobacter cloacae*, and *Candida albicans* [52]. In addition, certain starter cultures have shown a ropy (viscous) phenotype on solid media containing sugar and are mainly used to increase viscosity and prevent yogurt syneresis and gel fracture [53]. In the present study, in vitro analysis indicated that both UTNCys6-1 and L.LAC are slimy phenotype strains (Figure S4A) with similar growth in the tested sugar-containing media (Figure S4B). EPSS obtained from UTNCys6-1 cultured in MRSP medium demonstrated the greatest inhibitory activity (*p* < 0.05) against *Enterobacter hormechei* UTNB3Sh1 and *Listeria*, followed by *Salmonella* and *Staphylococcus* (Table 2, Figure S4C). Similarly, the EPSS derived from the reference strain L.LAC inhibited *Salmonella*, *S. aureus*, and *Enterobacter* but had less effect on the other pathogens examined. Although EPSQ retrieved from the MRSP liquid medium showed less inhibitory activity than its solid counterpart, the EPSQ activity of UTNCys6-1 towards *Listeria* was statistically significant (*p* < 0.05). EPSS and EPSQ obtained from both liquid and solid MRSD and MRSS showed comparable inhibitory activity. Exopolysaccharides are made up of polysaccharides, lipids, extracellular DNA, and non-carbohydrate substitutes (acetate, succinate, etc.) [52]. Their overall inhibitory capacity may vary depending on the substrate’s medium composition, the producer strain, and differences in the molecules that make up the composition. Further EPSS SDS-PAGE analysis showed that the EPS extracts of UTNCys6-1 recovered from MRSP had a larger band than their equivalents obtained from dextrose or sucrose. No such differences were observed in the protein profile of L.LAC (Figure S5). Taken together, the results showed that both PPE and EPSS and to a lesser extent EPSQ of UTNCys6-1 showed antilisterial activity; therefore, they are suitable candidates to be further tested in different food matrices susceptible to *Listeria* growth. However, both PPE and EPSs showed antimicrobial potency; their composition should be evaluated in conjunction with ex vitro assessment of inhibitory impact or probiotic features.

## 3. Materials and Methods

### 3.1. Bacterial Isolation and Selection

The bacterial strain was isolated from wild fruits of camu-camu (*Myrciaria dubia* H.B.K Mc Vaugh), originated from the Cuyabeno rainforest (Sucumbios Province, Ecuador), using the dilution plating method, and incubated on De Man, Rogosa, and Sharpe (MRS) agar (Difco, Detroit, MI, USA) at 37 °C for 72 h under anaerobic conditions. To choose the candidates, a series of tests including Gram staining, motility, indole and catalase production, spore formation, and gas generation from glucose were evaluated [7]. Additionally, the ability to survive various stressful conditions, including acidic pH (2.5–4.0), 0.3–1% bile, and high antibacterial capacity against food pathogens, were evaluated [7]. The isolate with the assigned code UTNCys6-1 was chosen for sequencing.

### 3.2. De Novo Assembly and Workflow Sequencing

An Illumina HiSeq X Ten platform was used for genome sequencing (Macrogen Inc.; Seoul, Korea). DNA/RNA was extracted from the sample using an Illumina DNA prep kit (Illumina Inc., San Diego, CA, USA). After quality control inspection, library construction was completed. According to the manufacturer’s recommended methodology, the sequencing library was created by randomly fragmenting a DNA or cDNA sample followed by 50 and 30 adapter ligations. The PCR-amplified adapter-ligated fragments were subsequently gel purified. For cluster generation, the library was loaded into a flow cell where fragments were captured on a lawn of surface-bound oligos complementary to the library adapters. Each fragment was amplified into distinct clonal clusters through bridge amplification or ExAmp cluster amplification (patterned flow cells) followed by sequencing, and the generated raw data were analyzed. The Illumina SBS technology makes use of a unique, terminator-based reversible technique to identify single bases as they are added to DNA template strands. As all 4 reversible, terminator-bound dNTPs are present during each sequencing cycle, natural competition minimizes the incorporation bias and greatly reduces raw error rates compared to other technologies. The result was highly accurate base-by-base sequencing that virtually eliminates sequence-context-specific errors, even within repetitive sequence regions and homopolymers. Sequencing data were converted into raw data for analysis, and the overall quality of reads generated by FastQC (v0.11.5, http://www.bioinformatics.babraham.ac.uk/projects/fastqc accessed on 6 March 2023), total base, total reads, GC content, and basic statistics were calculated. De novo assembly was performed with various k-mers using SPAdes 3.15.1 [54]. The best k-mer was selected based on various statistics from the assembly results (number of contigs, total base of contigs, N50, etc.) and the best assembled sequence set was determined. To assess the completeness of the genome assembly, BUSCO version 3.0 analysis was performed based on evolutionarily informed expectations of gene content from near-universal single-copy orthologs [55]. Analyses were performed by default using the eukaryote or bacterial DB. To determine the species to which each scaffold showed similarity, a BLAST analysis was conducted.

### 3.3. Typing and Species Relatedness

Average nucleotide identity (ANI) is a measure of nucleotide-level genomic similarity between the same taxon and species available in the NCBI database [56]. ANI analysis was performed with the taxon of the reference sequence (Taxon ID: 1358, *L. lactis* VKM B-1662) matched to contig 1 by BLASTN (custom assay project, Macrogen Inc.; Seoul, Korea). The top 5 closest genomes were detected. In addition, a circular map was generated by importing the FASTA sequences of UTNCys6-1 into the PROKSEE server [57]. In addition, genome sequence data were uploaded to the Type (Strain) Genome Server (TYGS) to conduct a complete genome-based taxonomic study [58].

### 3.4. General Genome Features, Gene Prediction and Functional Annotation

CDS, rRNA, tRNA/tmRNA, signal leader peptide, and noncoding RNA predictions were made as described [9]. Subsequent gene annotation was carried out with Prokka v1.14.5 [59]. Functional annotation was performed with InterProScan v.5.0 [60], which scores the sequences by family level and then checks them against the database registered with the signature of InterPro’s member databases, such as Pfam, Conserved Domain Database (CDD), and TIGRFAM (collection of manually curated protein families mainly focused on prokaryotic sequences) [61]. In addition, Evolutionary Genealogy of Genes: Non-supervised Orthologous Groups (EggNOG DB) was used for additional annotations [62]. psi-BLAST was employed to fit the predicted protein sequences with EggNOG DB.

### 3.5. In Silico Analysis

#### 3.5.1. Prediction of CRISPR Sequences, Prophages, ARGs, VFs, GIs, ISs and Pathogenicity

To identify CRISPR-Cas sequences and prophage sequences in bacterial genomes, CRISPRCasFinder (Crispr-Cas++1.1.2) and PHAge Search Tool Enhanced Release (PHASTER) were applied [63,64]. To detect components of virulence and antibiotic resistance, the Comprehensive Antibiotic Resistance Database (CARD v. 3.2.6) tool [65] and Resistance Gene Identifier (RGI v.6.0.1) (under perfect hit; rigorous hit alone; and perfect, strict, and loose hit criteria) were used [64]. To predict acquired antimicrobial resistance genes, the ResFinder 4.1 server was used with a selected % ID threshold of 90.00% and the selected minimum length of 60% and/or chromosomal mutations [66]. The web tool PlasmidFinder 2.0 [67] was used for the study of mobile elements, and the VFDB virulence component database [68] was used for the prediction of putative VFs. The web server IslandViewer 4 was used to predict GIs [69]. In addition, the ISfinder tool (ISsaga V.2.0) [70] was used to detect ISs. The PathogenFinder web server [71] was used to predict bacterial pathogenicity.

#### 3.5.2. Pangenome Analysis

To group genes that encode full protein sequences into a core (hard core and soft core) and accessory (shell and cloud) genome, Roary v3.12.0 [72] with the aligner MAFFT v7.427 was used [73]. Genome assembly data for the *Lactococcus* strains (10) used in this study were obtained from the NCBI online database (Table S6).

#### 3.5.3. Primary and Secondary Metabolites and Bacteriocin-Encoding Gene Prediction

FASTA contig input was imported into gutSMASH (Specialized Primary Metabolite Analysis from Anaerobic Bacteria) for the prediction of the primary metabolites, and antiSMASH version 6.0.1 (Antibiotic and Secondary Metabolites Shell) to predict secondary metabolites [74,75]. For the detection of biosynthetic gene clusters (BGCs) of antimicrobial compounds, the web tool BAGEL 4 was used [76]. The BGCs were contrasted with the reference strains *L. lactis* subsp. *lactis* ATCC11474 (L.LAC, ATCC genome webserver), *L. lactis* ATCC19435 (GCA_001456385.1), and *L. lactis* subsp. *lactis* KF282 (GCA_001456615.1).

### 3.6. In Vitro Analysis

#### 3.6.1. Physiological Characteristics and Antibiotic Susceptibility

Physiological characteristics (acid production, carbon source utilization, enzyme activity, and biochemical features) were determined using the BBL Crystal Anaerobe Gallery (cat # 245010, BD Company, Franklin Lakes, NJ, USA) in accordance with the manufacturer’s instructions. A nisin-derived reference *L. lactis* ATCC11474 strain [77] was used. Antibiotic susceptibility was determined using the MRS agar disk diffusion procedure according to Clinical and Laboratory Standards Institute (CLSI) guidelines [44]. Briefly, 100 μL of inoculum (10^7^–10^8^ CFU/mL) was streaked onto MRS plates. Commercial antibiotic disks of amoxicillin (AMX: 25 μg), ampicillin (AM: 10 μg), gentamicin (CN: 10 μg), kanamycin (K: 30 μg), amoxicillin/clavulanic acid (AMC: 20/10 μg), tetracycline (TE: 30 μg), and cefuroxime (CXM: 30 μg) (Merck, Rahway, NJ, USA) were plated on MRS agar plates and incubated at 37 °C for 48 h, and the diameter of each clear zone was measured in millimeters by scanning the plates with a microplate reader (SCAN500, Interscience, Saint Nom la Brétèche, France). *Escherichia coli* ATCC25922 was used as quality control. In addition, the E-test (Biomerioux, Marcy-l’Étoile, France) was used to confirm antibiotic resistance. The microbiological breakpoints reported by the FEEDAP standards were used to categorize lactobacilli as susceptible or resistant [44].

#### 3.6.2. Generation of EPSs

Using MRS agar containing 5% sucrose (MRSS), 5% dextrose (MRSD), and 5% panela (MRSP, unrefined sugar obtained from sugarcane), the presence of the UTNCys6-1 “ropy or slimy” appearance phenotype was evaluated in vitro after 72 h incubation at 37 °C [78]. Independently, cell biomass collected in sterile water was precipitated for 24 h under cooling (4 °C) with 2 volumes of ice-cold absolute ethanol and centrifuged (10,000× *g*, 20 min, 4 °C), and then the pellet was dissolved in sterile water, filtered, and further used in the antimicrobial assay. The filtered solution was annotated EPSS (EPSs recovered from solid MRS media containing the said sugars). In addition, an overnight culture of bacteria (1 × 10^8^ CFU/mL) was independently inoculated into MRS broth containing sugars as described above and incubated at 37 °C for 48 h. The bacterial culture was then removed from the extract by centrifugation (10,000× *g*, 20 min, 4 °C). EPSs were separated from the cell-free supernatant by the addition of ice-cold absolute ethanol (two volumes) and incubation for 2 days with refrigeration [79]. The precipitated EPSs from liquid culture (EPSQ) were centrifuged and dissolved in double distilled water and dialyzed using a Midi dialysis kit (cat # PURD10005-1KT, Sigma-Aldrich, St. Louis, MO, USA). The crude EPSQ was further purified by using a 10 kDa cut-off membrane (Vivaspin 500, Sartorius, Göttingen, Germany). A similar procedure was applied to the reference strain L.LAC. The final product was kept refrigerated until it was used in further antimicrobial testing experiments.

#### 3.6.3. PPE Extraction and Size Estimation Using Tricine-SDS-PAGE Analysis

PPE was obtained from 80% ammonium sulfate precipitation of the cell-free supernatant extracted from the overnight growth culture of the UTNCys6-1 strain and recovered by centrifugation at 13,000× *g* for 20 min (4 °C) followed by filtration using a 0.22 μm porosity syringe filter (# STF020025H, ChemLab Group, Vernon, FL, USA), incubation at (4 °C) for 24 h, and centrifugation at 13,000× *g* for 30 min [7]. The final precipitated molecules were dissolved in ammonium acetate (25 mM, pH 6.0). The same procedure was used to generate PPE from the L.LAC reference strain. The samples were stored at −20 °C before being used in the antimicrobial assay. The broad-range protein molecular marker (Cat. # V8491, Promega, Madison, WI, USA) was used to estimate the molecular size. Commercial nisin from *L. lactis* at 2.5% (balance sodium chloride) (50 mg/mL) was incorporated as a control. The precast acrylamide gels (4–20%) and a mini-vertical electrophoresis system (Expedeon Ltd., Abcam, Cambridge, MA, USA) were employed. The gel was stained with InstantBlue ready-to-use stain (Expedeon, Abcam, Cambridge, MA, USA) according to the manufacturer’s instructions.

#### 3.6.4. In Vitro Antimicrobial Activity of PPE and EPSs

The antimicrobial activity of PPE, EPSS, and EPSQ obtained from UTNCys6-1 and L.LAC strains was evaluated against *S. aureus* ATCC1026 (MRSA strain), *S. aureus* ATCC43300 (MRSA strain), *L. monocytogenes* ATCC19115, *S. enterica* subsp. *enterica* ATCC51741, *E. coli* ATCC25922, and *E. hormechei* UTNB3Sh1 (a laboratory multidrug-resistant strain isolated from natural juice) using a standard well-assay method. In brief, each indicator strain (100µL) was cultured in an appropriate broth medium (7 logCFU/mL) and mixed with 3.5 mL of MRS soft agar (0.75%). It was then overlaid on nutrient agar plates and incubated at 37 °C for 2 h. The PPE, EPSS, and EPSQ (100 µL) were transferred onto the reaction wells (6 mm) with overlaid agar and incubated at 37 °C, and the presence of an inhibition zone was evaluated at 48 h. Broth MRS medium was used as a negative control. The experiments were carried out in triplicate, and the mean value of the inhibition zone was determined by scanning the plates with a microplate reader (SCAN500, Interscience, Saint Nom la Brétèche, France).

## 4. Conclusions

To our knowledge, this study is the first study characterizing the complete genome of a lactococcal strain isolated from wild Amazonian camu-camu fruits. The isolate was assigned as *Lactococcus lactis* UTNCys6-1. Based on in silico genomic characterization and in vitro analysis, UTNCys6-1 is a harmless, non-pathogenic strain that secretes several functional metabolites with biotechnological potential. However, four BGCs, including the most prevalent lantibiotic nisin Z, have been detected in the genome, indicating that UTNCys6-1 is a possible nisin Z-producing strain. In vitro research validated the strain’s ability to metabolize several sugar-containing substrates, agreeing with in silico metabolic profile analysis, suggesting the strength of its niche adaptation, which might include a food-based matrix. Pangenome analysis revealed the presence of a sucrose transporter of the SemiSWEET family in the genome of the UTNCys6-1 strain only; its functionality should be investigated. Preliminary research indicates that UTNCys6-1 produces inhibitory metabiotic compounds such as PPE and EPSs that are effective against multidrug-resistant *Listeria* spp. and *Staphylococcus* spp. Studies in vitro are currently being undertaken to assess its promising inhibitory strength against *Listeria* and *Staphylococcus* ex vitro in various food matrices. In vitro and ex vitro study on the probiotic potential is still needed to fully utilize its technical potential for use in the agri-food and pharmaceutical industries.

## Figures and Tables

**Table 1:** MGC regions predicted in the UTNCys6-1 genome.

Primary Metabolites			
Contig. Region	Type	Location (Length)	Most Similar Gene Cluster ClusterBlast */KnownCluster Blast Gene Similarity (%)
1.1	GR_AA_metabolism	89,383–125,845 nt	Not match
		(total: 36,463 nt)	
1.2	TPP_fatty_acids	253,778–280,874 nt	Not match
		(total: 27,097 nt)	
1.3	TPP_AA_metabolism	719,434–745,727 nt	Threonine to propionate (20%)
		(total: 26,294 nt)	
3.1	Arginine2_Hcarbonate	85,098–111,226 nt	Arginine to hydrogen carbonate of *P. aeruginosa* (100%)
		(total: 26,129 nt)	
4.1	Others_HGD_unassigned	36,177–60,487 nt	Not match
		(total: 24,311 nt)	
**Secondary metabolites**			
1.1	Beta lactone	93,313–125,845 nt	100/nisin A
		(total: 32,533 nt)	
1.2	Lanthipeptide-class I	127,000–153,031 nt	
		(total: 26,032 nt)	
1.3	T3PKS	1,112,828–1,153,982 nt	
		(total: 41,155 nt)	
2.1	RaS-RiPP	31,471–53,861 nt	Not match
		(total: 22,391 nt)	
5.1	RaS-RiPP	12,025–34,680 nt	Not match
		(total: 22,656 nt)	

Contig region: the number of the regions of the primary and secondary metabolites detected in the Cys6-1 genome; Type: the metabolite cluster name; * % similarity with several *Lactococcus* strains from database; nt, nucleotides.

**Table 2:** Inhibitory activity of PPE, EPSQ, and EPSS obtained from *L. lactis* UTNCys6-1 and *L. lactis* ATCC11474 (L. LAC).

Indicator Strain	Average Diameter of the Inhibition Zone (mm)					
	PPE		EPSQ		EPSS	
	UTNCys6-1	L. LAC	UTNCys6-1	L. LAC	UTNCys6-1	L. LAC
*S. aureus* ATCC1026	9.33 ± 0.58 ^Db^	10.33 ± 0.58 ^Aa^	9.33 ± 0.58 ^Bb^	9.33 ± 0.58 ^Bb^	9.17 ± 0.29 ^Cb^	9.17 ± 0.29 ^Cb^
*S. aureus* ATCC43300	9.33 ± 0.58 ^Dd^	10.33 ± 0.58 ^Ac^	9.33 ± 0.58 ^Bd^	9.33 ± 0.58 ^Bd^	12.33 ± 0.58 ^Bb^	13.17 ± 0.29 ^Ba^
*L. monocytogenes* ATCC19115	18.03 ± 0.05 ^Aa^	9.33 ± 0.58 ^Be^	11.33 ± 0.58 ^Ac^	10.17 ± 0.29 ^Ad^	12.67 ± 0.58 ^Bb^	9.33 ± 0.58 ^Ce^
*S. enterica* subsp. *enterica* ATCC51741	12.33 ± 0.94 ^Ca^	9.33 ± 0.58 ^Bb^	8.33 ± 0.58 ^Bc^	8.33 ± 0.58 ^Cc^	12.33 ± 0.94 ^Ba^	12.33 ± 0.94 ^Ba^
*E. coli* ATCC25922	9.33 ± 0.58 ^Db^	10.33 ± 0.58 ^Aa^	8.33 ± 0.58 ^Bc^	8.33 ± 0.58 ^Cc^	9.33 ± 0.58 ^Cb^	9.33 ± 0.58 ^Cb^
*E. hormechei* UTNB3Sh1	14.67 ± 0.94 ^Ba^	9.33 ± 0.58 ^Bd^	8.33 ± 0.58 ^Be^	8.33 ± 0.58 ^Ce^	14.33 ± 0.58 ^Ab^	13.67 ± 0.58 ^Ac^
MRS broth (negative control)	6.01 ± 0.20 *^Ea^	6.01 ± 0.20 *^Ca^	6.01 ± 0.20 *^Ca^	6.01 ± 0.20 *^Da^	6.01 ± 0.20 *^Da^	6.01 ± 0.20 *^Da^

* Diameter of the agar well/no inhibition. The mean (±standard deviation) of the diameter of the inhibition zone (mm) is shown. Values with different letters are significantly different *p* < 0.05. Values in the same column that are statistically different (*p* < 0.05) are indicated with capital letters. Values in the same row with lowercase letters are significantly different within the strain producer category (PPE, EPSQ, and EPSS). PPE: peptide–protein extract; EPSQ: EPSs obtained from liquid MRSP culture; EPSS: EPSs obtained from solid MRSP; MRSP: MRS with 5% panela.

## Data Availability

The datasets presented in this study can be found in online repositories. The names of the repository/repositories and accession number(s) can be found in the article/Supplementary Materials.

## Supplementary Materials

The following supporting information can be downloaded at: https://www.mdpi.com/article/10.3390/ijms24076127/s1.
