# Supplementary material for: Metabiotics Signature through Genome Sequencing and In Vitro Inhibitory Assessment of a Novel Lactococcus lactis Strain UTNCys6-1 Isolated from Amazonian Camu-Camu Fruits
Source: Int J Mol Sci. 2023 Mar 24;24(7):6127. doi: 10.3390/ijms24076127 (PMC10094308; doi:10.3390/ijms24076127)

Metabiotics Signature through Genome Sequencing and In Vitro Inhibitory Assessment of a Novel *Lactococcus lactis* Strain UTNCys6-1 Isolated from Amazonian Camu-Camu Fruits

Gabriela N. Tenea
Biofood and Nutraceutics Research and Development Group, Faculty of Engineering in Agricultural and Environmental Sciences, Universidad Técnica del Norte, Av. 17 de Julio s-21, Barrio El Olivo, Ibarra 100150, Ecuador; gntenea@utn.edu.ec or gtenea@hotmail.com

Figure S1. ANI analysis results. A. Percent identity heatmap. The cells corresponding to an ANI value of 95% and higher are stained red, indicating that the corresponding strains belong to the same species. The dendrograms (in green; above and on the left side), which were constructed by the simple linkage of the ANIm (ANI with MUMmer) percentage identities, correspond to the results of the clustering of the ANI values between the used strains. **B).** Alignment coverage heatmap. The isolates and species assignments as indicated at source are given as row and column labels. Cells in the heatmap corresponding to 75% coverage or greater are colored red. Color intensity fades as the comparisons approach 50% coverage. The dendrograms (in green; above and on the left side) of the heatmap correspond to strains assignments for each isolate in the analysis. ANI: Average Nucleotide Identity

A.

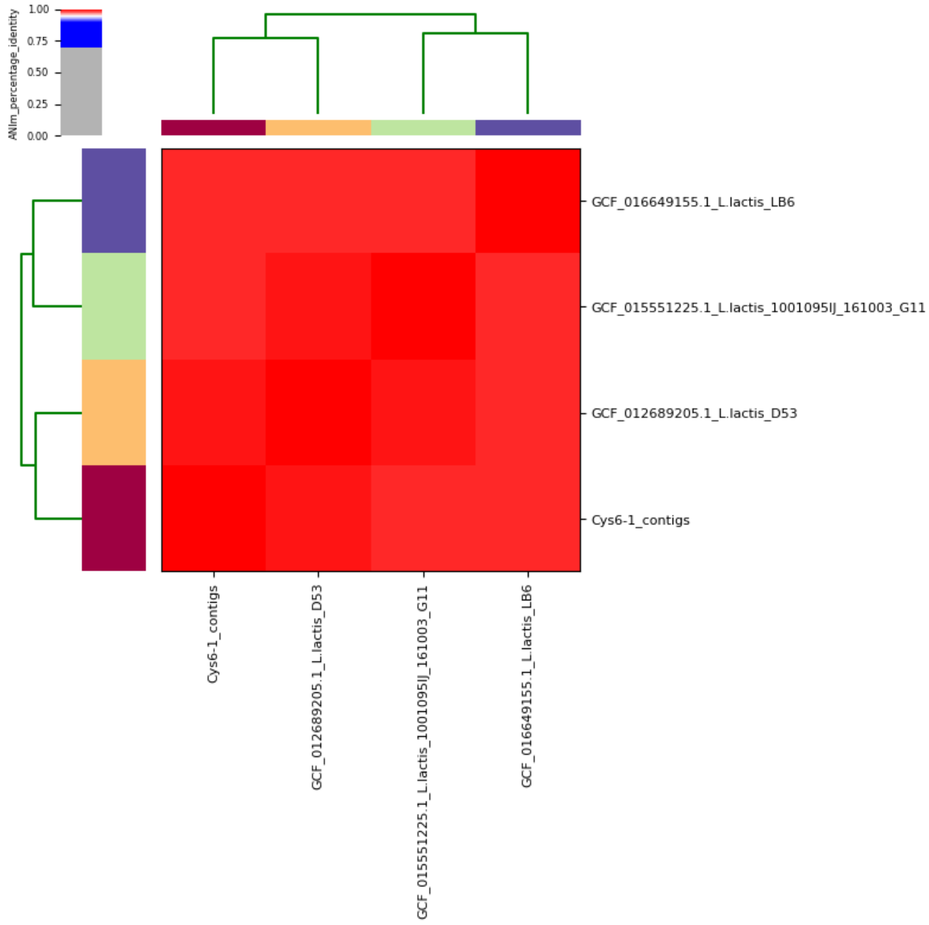

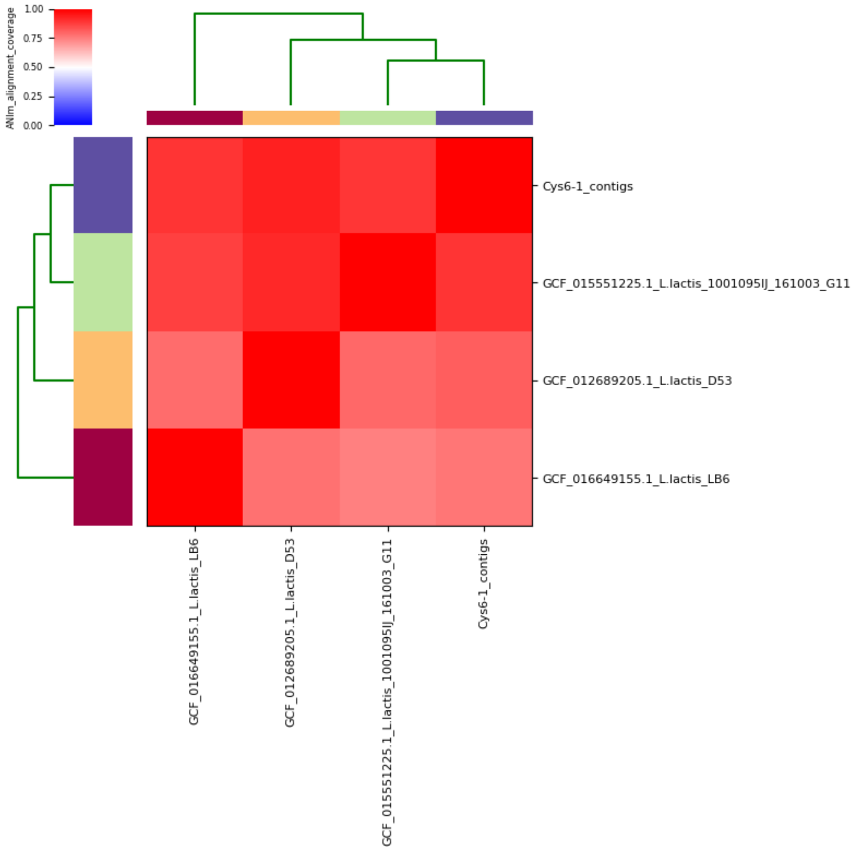
ANIm_alignment_coverage
1.00
0.75
0.50
0.25
0.00
Cys6-1_contigs
GCF_015551225.1_L.lactis_1001095IJ_161003_G11
GCF_012689205.1_L.lactis_D53
GCF_016649155.1_L.lactis_LB6
GCF_016649155.1_L.lactis_LB6
GCF_012689205.1_L.lactis_D53
GCF_015551225.1_L.lactis_1001095IJ_161003_G11
Cys6-1_contigs

B.

Figure S2. Genes annotated with CARD protein ID and grouped by Drug Class. The number of genes in each category is shown.

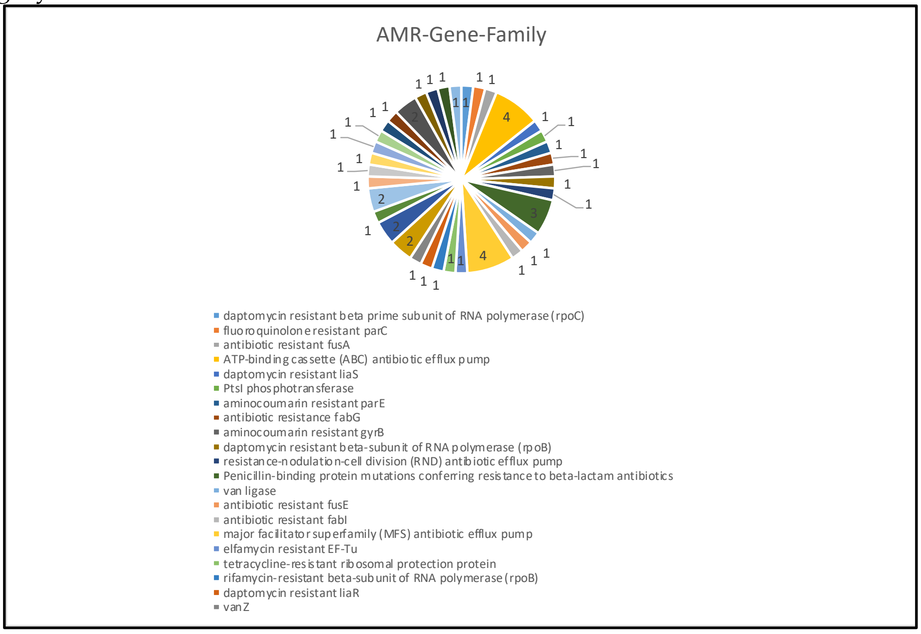

Figure S3. Metabolic profile based on the BBL Crystal miniaturized test. Black color indicated positive (metabolized the substrate), white color negative (do not metabolize the substrate).

**Metabilite**	***L. lactis* UTNCys6-1**	***L. lactis* ATCC11474**
**4MU-β-D-glucoside FGC**	■	■
**L-valine / AMC FVA**		■
**L-phenylalanine AMC FPH**		
**4MU-α-D-glucoside FGS**	■	■
**L-pyroglutamic acid-AMC FPY**		■
**L-tryptophan FTR**	■	■
**L-arginine -AMC FAR**	■	■
**4MU-N-acetyl-β-D-glucosaminide FGA**	■	■
**4MU-phosphate - FHO**		■
**4MU-β-D-glucuronide - FGN**		
**L-isoleucine -AMC FIS**		■
**Trehalose / TRE**	■	■
**Lactose / LAC**	■	■
**Methyl-α & β-glucoside MAB**	■	■
**Sucrose / SUC**	■	■
**Mannitol / MNT**	■	
**Maltotriose / MTT**	■	
**Arabinose / ARA**	■	
**Glycerol / GLR**	■	
**Fructose / FRU**	■	
**p-n-p-β-D-glucoside / BGL**		
**p-n-p-β-D-cellobioside / PCE**	■	
**Proline & Leucine-p-nitroanilide PLN**	■	
**p-n-p-phosphate / Phosphat / fosfato PHO**		
**p-n-p-α-D-maltoside / maltósido PAM**	■	
**ONPG & p-n-p-α D-galactoside / PGO**		
**Urea / URE**		
**Esculin / ESC**	■	■
**Arginine / ARG**		
**Fluorescent negative control / FCT –**		

Supplementary Figure 4. A. Slimy phenotype of EPS produced by *L. lactis* UTNCys6-1 (Cys6-1) and *L. lactis* ATCC11454 (L.LAC) at 24 h and 72h of incubation at 37 °C. **B.** Viable cells profile in medium containing sugars; **C.** Zone of inhibition formed by EPSS against *Listeria monocytogenes* (a) and *Enterobacter hormechei* UTNB3Sh1 (b). Legend: MRSS: MRS with 5% sucrose; MRSP: MRS with 5% panela; MRSD: MRS with 5% dextrose; panel B: serial dilution $1:10^6$; panel C: 1, 2, 3: independent repetitions.

A.

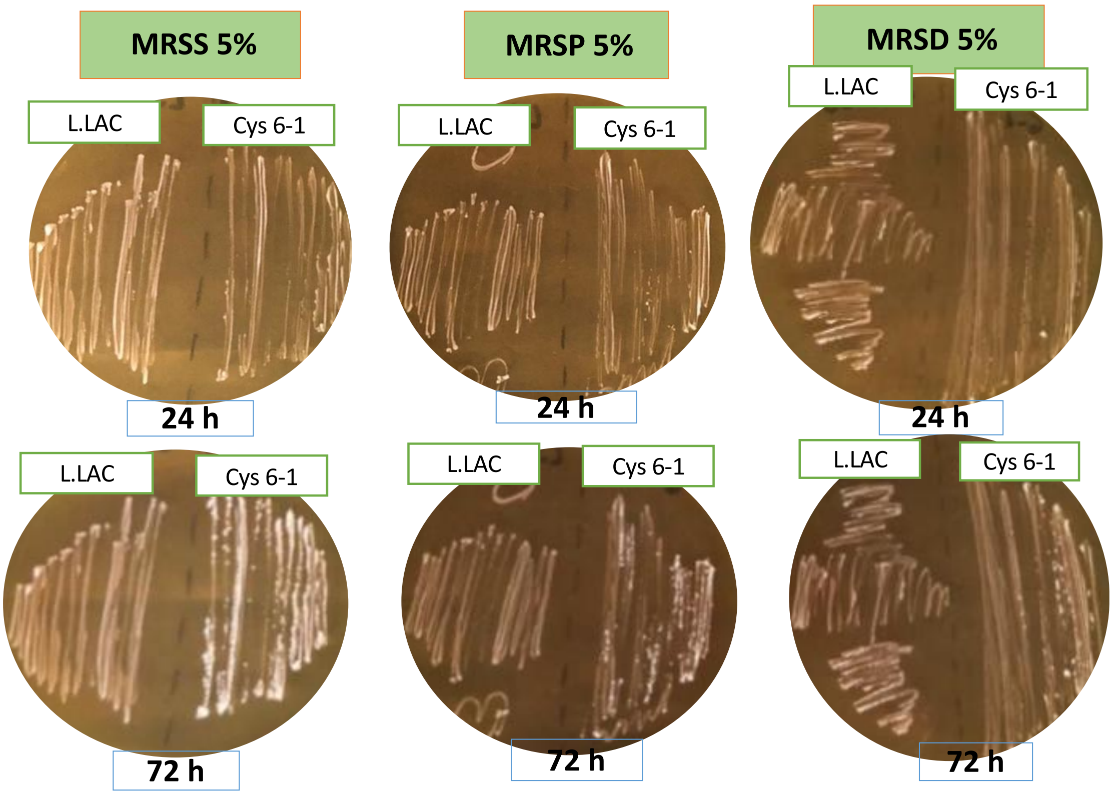

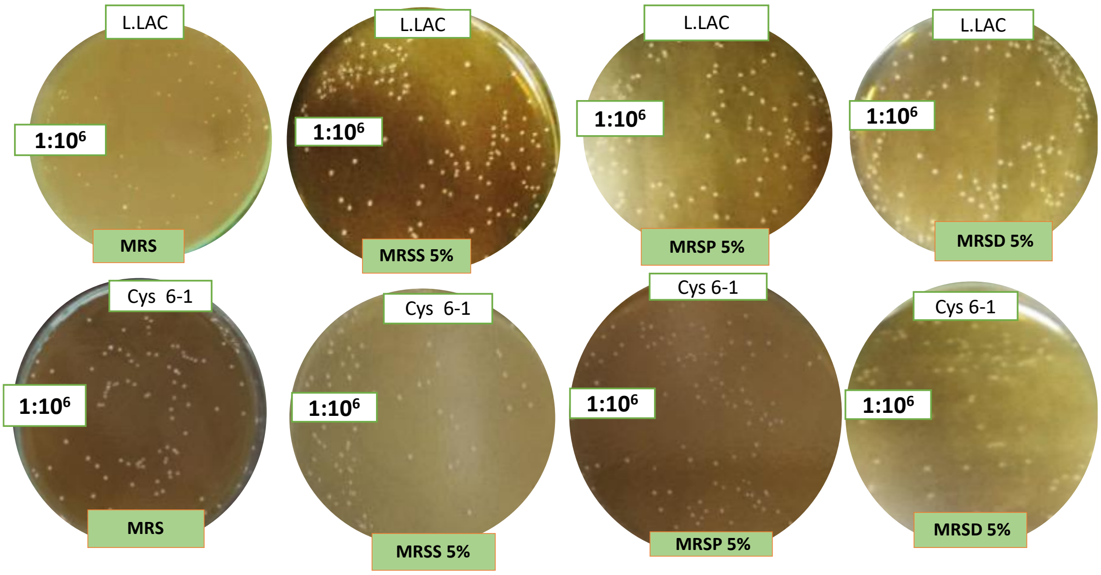

B.

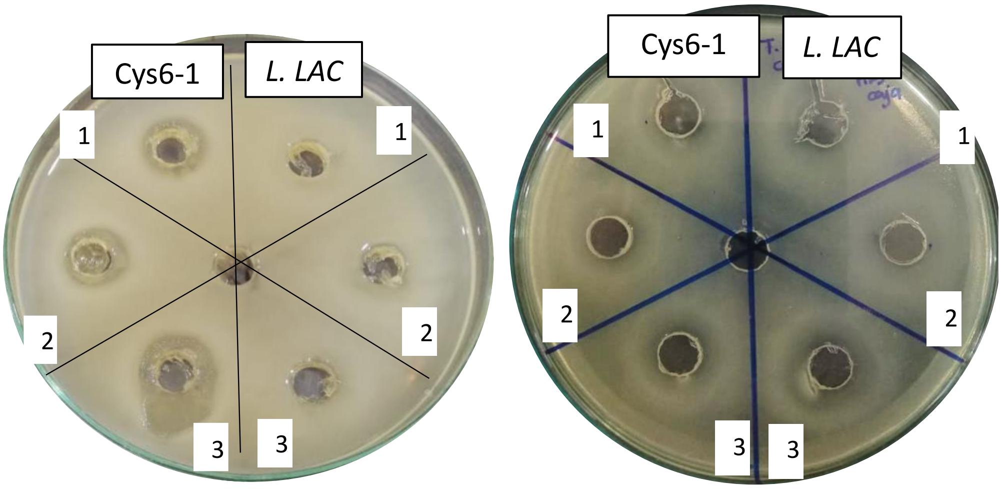

a) b)

C.

Figure S5. Tricine-SDS-PAGE profile of EPSS. Legend: M-molecular marker (Promega). 1, 3, 5: EPSS obtained from *L. lactis* ATCC11474 grown in MRSS, MRSD, and MRSP agar medium. 2, 4, 6: EPSS obtained from *L. lactis* UTNCys6-1 grown in MRSS, MRSD, and MRSP agar medium.

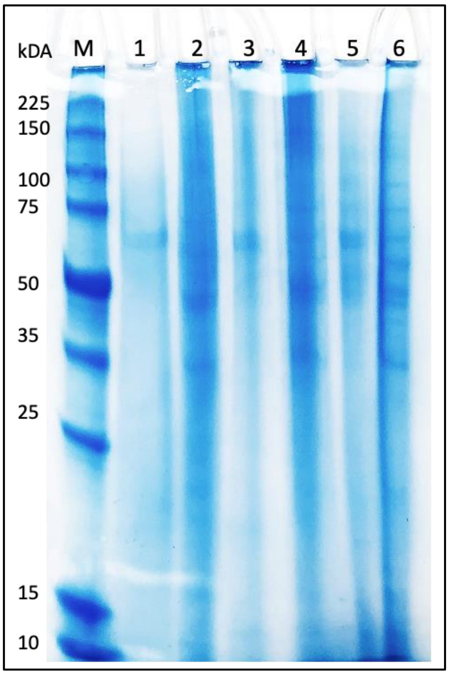

Supplement: Supplementary file 1 [file ijms-24-06127-s001.zip › Supplementary Figures.pdf]
