# Supplementary material for: Metabiotics Signature through Genome Sequencing and In Vitro Inhibitory Assessment of a Novel Lactococcus lactis Strain UTNCys6-1 Isolated from Amazonian Camu-Camu Fruits
Source: Int J Mol Sci. 2023 Mar 24;24(7):6127. doi: 10.3390/ijms24076127 (PMC10094308; doi:10.3390/ijms24076127)
Supplement: Supplementary file 1 [file ijms-24-06127-s001.zip › Supplementary Tables.pdf]

Metabiotics Signature through Genome Sequencing and In Vitro Inhibitory Assessment of a Novel *Lactococcus lactis* Strain UTNCys6-1 Isolated from Amazonian Camu-Camu Fruits

Gabriela N. Tenea

Biofood and Nutraceutics Research and Development Group, Faculty of Engineering in Agricultural and Environmental Sciences, Universidad Técnica del Norte, Av. 17 de Julio s-21, Barrio El Olivo, Ibarra 100150, Ecuador; gntenea@utn.edu.ec or gtenea@hotmail.com

Table S1. De novo assembly summary

Assembly Method	SPAdes 3.13.0
Contigs	7
Total contig bases	2,331,682
N50	1,520,392
GC content(%)	34.88
Estimated genome size (bp)	2,226,248
Sequencing depth	142

Contigs: The number of contigs assembled
Total bases of contigs : The total length of contigs
N50 : 50% of all contig bases come from contigs longer than this value
GC content: The percentage of guanine-cytosine base pairs
Genome size: The total of the genome in bp
Sequencing Depth : Average mapping depth

Table S2. Summary of Prophage regions identified in the UTNCys6-1 genome

Contig	Region	Region length	Completeness	Score	# Total proteins	Region position	Most common Phage	GC%
1	1	10.8 kb	incomplete	20	17	73840-84652	PHAGE_Lactoc_bIL286_ NC_002667(3)	34.30
1	2	18.7 kb	incomplete	30	14	1168206-1186978	PHAGE_Lactoc_TP901_1_NC_002747(2)	35.14
2	1	6.4 kb	incomplete	20	7	367721-374207	PHAGE_Paenib_Yerffej_NC_048714(1)	36.09
3	1	20.7 kb	incomplete	20	30	134918-155691	PHAGE_Lactob_bIL3010_NC_002669(8)	40.79

Legend: region: the number assigned to the region; region length: the length of the sequence in that region; completeness: a prediction of whether the region contains a intact or incomplete prophage based on the criteria: intact (score > 90%); questionable (score 70-90); incomplete (score < 70%); score: the score of the region based on the mentioned criteria; # total proteins: the number of ORFs present in the region; Region position: the start and end positions of the region on the bacterial chromosome; Most common phage: the phages (s) with the highest number of proteins most similar to those in the region; GC%: the percentage of GC nucleotides of the region.

Table S3. Genomic islands detected in the UTNCys6-1 genome

Island start	Island end	Length	Method	Gene ID	Locus	Gene start	Gene end	Strand	Product
591735	643354	51619	Predicted by at least one method		Cys6-1_00136	591735	592766	1	hypothetical protein
591735	643354	51619	Predicted by at least one method	icaA	Cys6-1_00137	592771	593991	1	Poly-beta-1,6-N-acetyl-D-glucosamine synthase
591735	643354	51619	Predicted by at least one method		Cys6-1_00138	593994	594254	1	hypothetical protein
591735	643354	51619	Predicted by at least one method	icaB	Cys6-1_00139	594264	595157	1	Poly-beta-1,6-N-acetyl-D-glucosamine N-deacetylase
591735	643354	51619	Predicted by at least one method		Cys6-1_00140	595184	596638	1	hypothetical protein
591735	643354	51619	Predicted by at least one method	brnQ	Cys6-1_00141	596716	598086	-1	Branched-chain amino acid transport system 2 carrier protein
591735	643354	51619	Predicted by at least one method	yjdJ	Cys6-1_00142	598323	598595	-1	putative protein YjdJ
591735	643354	51619	Predicted by at least one method		Cys6-1_00143	598595	598867	-1	hypothetical protein
591735	643354	51619	Predicted by at least one method	nisZ	Cys6-1_00144	600280	600453	1	Lantibiotic nisin-Z
591735	643354	51619	Predicted by at least one method	nisB	Cys6-1_00145	600561	603542	1	Nisin biosynthesis protein NisB
591735	643354	51619	Predicted by at least one method	msbA	Cys6-1_00146	603553	605355	1	Lipid A export ATP-binding/permease protein MsbA
591735	643354	51619	Predicted by at least one method	nisC	Cys6-1_00147	605348	606592	1	Nisin biosynthesis protein NisC
591735	643354	51619	Predicted by at least one method	nisI	Cys6-1_00148	606589	607326	1	Nisin immunity protein
591735	643354	51619	Predicted by at least one method	nisP	Cys6-1_00149	607328	609376	1	Nisin leader peptide-processing serine protease NisP
591735	643354	51619	Predicted by at least one method	arlR_1	Cys6-1_00150	609445	610131	1	Response regulator ArlR

591735	643354	51619	Predicted by at least one method	sasA_1	Cys6-1_00151	610124	611467	1	Adaptive-response sensory-kinase SasA
591735	643354	51619	Predicted by at least one method	yxlF	Cys6-1_00152	611566	612243	1	putative ABC transporter ATP-binding protein YxlF
591735	643354	51619	Predicted by at least one method		Cys6-1_00153	612245	612973	1	hypothetical protein
591735	643354	51619	Predicted by at least one method		Cys6-1_00154	612960	613604	1	hypothetical protein
591735	643354	51619	Predicted by at least one method		Cys6-1_00155	614738	615448	1	hypothetical protein
591735	643354	51619	Predicted by at least one method		Cys6-1_00156	615493	616548	1	hypothetical protein
591735	643354	51619	Predicted by at least one method	bcsA	Cys6-1_00157	616603	618582	1	Cellulose synthase catalytic subunit [UDP-forming]
591735	643354	51619	Predicted by at least one method	degA_1	Cys6-1_00158	618679	619635	-1	HTH-type transcriptional regulator DegA
591735	643354	51619	Predicted by at least one method	scrB	Cys6-1_00159	619632	621050	-1	Sucrose-6-phosphate hydrolase
591735	643354	51619	Predicted by at least one method		Cys6-1_00160	621228	623180	1	hypothetical protein
591735	643354	51619	Predicted by at least one method	gmuE_1	Cys6-1_00161	623193	624065	1	Putative fructokinase
591735	643354	51619	Predicted by at least one method	hsrA_1	Cys6-1_00162	624527	625894	-1	putative transport protein HsrA
591735	643354	51619	Predicted by at least one method	ybbH_1	Cys6-1_00163	625942	626763	-1	putative HTH-type transcriptional regulator YbbH
591735	643354	51619	Predicted by at least one method	rbsK/rbiA	Cys6-1_00164	627100	627627	1	Bifunctional ribokinase/ribose-5-phosphate isomerase A
591735	643354	51619	Predicted by at least one method	iolU_1	Cys6-1_00165	627600	628577	1	scyllo-inositol 2-dehydrogenase (NADP(+)) IolU
591735	643354	51619	Predicted by at least one method		Cys6-1_00166	628823	629308	-1	hypothetical protein
591735	643354	51619	Predicted by at least one method		Cys6-1_00167	629667	630503	1	hypothetical protein
591735	643354	51619	Predicted by at least one method		Cys6-1_00168	631207	631494	1	hypothetical protein

591735	643354	51619	Predicted by at least one method	hin	Cys6-1_00169	631753	632307	-1	DNA-invertase hin
591735	643354	51619	Predicted by at least one method		Cys6-1_00170	632569	633021	-1	hypothetical protein
591735	643354	51619	Predicted by at least one method		Cys6-1_00171	633484	634089	-1	hypothetical protein
591735	643354	51619	Predicted by at least one method		Cys6-1_00172	634465	634656	-1	hypothetical protein
591735	643354	51619	Predicted by at least one method	rhaS_1	Cys6-1_00173	635546	636601	-1	HTH-type transcriptional activator RhaS
591735	643354	51619	Predicted by at least one method	xylP	Cys6-1_00174	636752	638128	1	Isoprimeverose transporter
591735	643354	51619	Predicted by at least one method		Cys6-1_00175	638125	639375	1	hypothetical protein
591735	643354	51619	Predicted by at least one method		Cys6-1_00176	639392	640969	1	hypothetical protein
591735	643354	51619	Predicted by at least one method	bglK_1	Cys6-1_00177	640970	641875	1	Beta-glucoside kinase
591735	643354	51619	Predicted by at least one method	bglA_1	Cys6-1_00178	641897	643354	1	6-phospho-beta-glucosidase BglA
1201794	1211161	9367	Predicted by at least one method	topA	Cys6-1_00700	1201794	1203926	1	DNA topoisomerase 1
1201794	1211161	9367	Predicted by at least one method	trmFO	Cys6-1_00701	1203998	1205341	1	Methylenetetrahydrofolate--tRNA-(uracil-5-)-methyltransferase TrmFO
1201794	1211161	9367	Predicted by at least one method	xerS_1	Cys6-1_00702	1205386	1206456	-1	Tyrosine recombinase XerS
1201794	1211161	9367	Predicted by at least one method		Cys6-1_00703	1206937	1207224	-1	hypothetical protein
1201794	1211161	9367	Predicted by at least one method	xerS_2	Cys6-1_00704	1207307	1208362	-1	Tyrosine recombinase XerS
1201794	1211161	9367	Predicted by at least one method		Cys6-1_00705	1209192	1209587	-1	hypothetical protein
1201794	1211161	9367	Predicted by at least one method		Cys6-1_00706	1209825	1210631	-1	hypothetical protein
1201794	1211161	9367	Predicted by at least one method		Cys6-1_00707	1210679	1211161	-1	hypothetical protein

1201794	1211161	9367	Predicted by at least one method		Cys6-1_00708	1211151	1212827	-1	hypothetical protein
1223353	1228766	5413	Predicted by at least one method		Cys6-1_00716	1223353	1225290	-1	hypothetical protein
1223353	1228766	5413	Predicted by at least one method		Cys6-1_00718	1227591	1228766	-1	hypothetical protein
1223353	1228766	5413	Predicted by at least one method		Cys6-1_00719	1228744	1230033	-1	hypothetical protein
1227591	1249990	22399	Predicted by at least one method		Cys6-1_00718	1227591	1228766	-1	hypothetical protein
1227591	1249990	22399	Predicted by at least one method		Cys6-1_00719	1228744	1230033	-1	hypothetical protein
1227591	1249990	22399	Predicted by at least one method		Cys6-1_00720	1230092	1231324	-1	hypothetical protein
1227591	1249990	22399	Predicted by at least one method		Cys6-1_00721	1231294	1231704	-1	hypothetical protein
1227591	1249990	22399	Predicted by at least one method	purK_1	Cys6-1_00722	1231724	1233001	-1	N5-carboxyaminoimidazole ribonucleotide synthase
1227591	1249990	22399	Predicted by at least one method	ldcA	Cys6-1_00723	1233123	1234070	-1	Murein tetrapeptide carboxypeptidase
1227591	1249990	22399	Predicted by at least one method	asnS_1	Cys6-1_00724	1234089	1235087	-1	Asparagine--tRNA ligase
1227591	1249990	22399	Predicted by at least one method	ubiE_2	Cys6-1_00725	1235122	1235937	-1	Ubiquinone/menaquinone biosynthesis C-methyltransferase UbiE
1227591	1249990	22399	Predicted by at least one method		Cys6-1_00726	1236371	1237285	1	hypothetical protein
1227591	1249990	22399	Predicted by at least one method		Cys6-1_00727	1237441	1237962	-1	hypothetical protein
1227591	1249990	22399	Predicted by at least one method		Cys6-1_00728	1237949	1238812	-1	hypothetical protein
1227591	1249990	22399	Predicted by at least one method	ltrA	Cys6-1_00729	1239244	1241043	-1	Group II intron-encoded protein LtrA
1227591	1249990	22399	Predicted by at least one method		Cys6-1_00730	1241581	1241979	-1	hypothetical protein
1227591	1249990	22399	Predicted by at least one method		Cys6-1_00731	1242029	1242361	-1	hypothetical protein

1227591	1249990	22399	Predicted by at least one method		Cys6-1_00732	1242539	1242628	-1	hypothetical protein
1227591	1249990	22399	Predicted by at least one method		Cys6-1_00733	1242659	1242979	-1	hypothetical protein
1227591	1249990	22399	Predicted by at least one method		Cys6-1_00734	1243368	1244282	-1	hypothetical protein
1227591	1249990	22399	Predicted by at least one method	hchA	Cys6-1_00735	1244283	1244960	-1	Molecular chaperone Hsp31 and glyoxalase 3
1227591	1249990	22399	Predicted by at least one method		Cys6-1_00736	1244957	1245274	-1	hypothetical protein
1227591	1249990	22399	Predicted by at least one method	ceo	Cys6-1_00737	1245749	1246678	-1	N(5)-(carboxyethyl)ornithine synthase
1227591	1249990	22399	Predicted by at least one method		Cys6-1_00738	1247055	1247606	-1	Stress response regulator gls24
1227591	1249990	22399	Predicted by at least one method		Cys6-1_00739	1247627	1247815	-1	hypothetical protein
1227591	1249990	22399	Predicted by at least one method		Cys6-1_00740	1247825	1248385	-1	hypothetical protein
1227591	1249990	22399	Predicted by at least one method		Cys6-1_00741	1248415	1248654	-1	hypothetical protein
1227591	1249990	22399	Predicted by at least one method	dkgB	Cys6-1_00742	1249148	1249990	1	2,5-diketo-D-gluconic acid reductase B
1621554	1640004	18450	Predicted by at least one method	arlR_2	Cys6-1_01063	1621554	1622246	-1	Response regulator ArlR
1621554	1640004	18450	Predicted by at least one method		Cys6-1_01064	1622402	1622932	-1	hypothetical protein
1621554	1640004	18450	Predicted by at least one method	rpmE2	Cys6-1_01065	1623063	1623308	-1	50S ribosomal protein L31 type B
1621554	1640004	18450	Predicted by at least one method		Cys6-1_01066	1623464	1623928	-1	hypothetical protein
1621554	1640004	18450	Predicted by at least one method		Cys6-1_01067	1624010	1624372	-1	hypothetical protein
1621554	1640004	18450	Predicted by at least one method		Cys6-1_01068	1624857	1625387	-1	hypothetical protein
1621554	1640004	18450	Predicted by at least one method	ypbG	Cys6-1_01069	1625448	1626284	-1	putative protein YpbG

1621554	1640004	18450	Predicted by at least one method		Cys6-1_01070	1626935	1627330	1	hypothetical protein
1621554	1640004	18450	Predicted by at least one method		Cys6-1_01071	1627999	1628268	-1	hypothetical protein
1621554	1640004	18450	Predicted by at least one method		Cys6-1_01072	1628589	1628837	1	hypothetical protein
1621554	1640004	18450	Predicted by at least one method		Cys6-1_01073	1629847	1630218	1	hypothetical protein
1621554	1640004	18450	Predicted by at least one method		Cys6-1_01074	1630468	1630653	-1	hypothetical protein
1621554	1640004	18450	Predicted by at least one method		Cys6-1_01076	1630891	1631169	-1	hypothetical protein
1621554	1640004	18450	Predicted by at least one method		Cys6-1_01080	1632233	1633018	-1	hypothetical protein
1621554	1640004	18450	Predicted by at least one method		Cys6-1_01081	1633075	1633293	-1	hypothetical protein
1621554	1640004	18450	Predicted by at least one method		Cys6-1_01082	1633306	1633527	-1	hypothetical protein
1621554	1640004	18450	Predicted by at least one method		Cys6-1_01083	1633543	1634292	-1	hypothetical protein
1621554	1640004	18450	Predicted by at least one method		Cys6-1_01084	1634395	1635126	-1	hypothetical protein
1621554	1640004	18450	Predicted by at least one method		Cys6-1_01085	1635605	1635844	-1	hypothetical protein
1621554	1640004	18450	Predicted by at least one method		Cys6-1_01086	1635962	1636231	1	hypothetical protein
1621554	1640004	18450	Predicted by at least one method		Cys6-1_01087	1636352	1636507	1	hypothetical protein
1621554	1640004	18450	Predicted by at least one method		Cys6-1_01088	1636727	1637113	1	hypothetical protein
1621554	1640004	18450	Predicted by at least one method		Cys6-1_01089	1637290	1637853	1	hypothetical protein
1621554	1640004	18450	Predicted by at least one method		Cys6-1_01090	1637883	1637981	-1	hypothetical protein
1621554	1640004	18450	Predicted by at least one method		Cys6-1_01091	1637990	1638232	-1	hypothetical protein

1621554	1640004	18450	Predicted by at least one method		Cys6-1_01092	1638204	1638407	-1	hypothetical protein
1621554	1640004	18450	Predicted by at least one method		Cys6-1_01093	1638870	1639016	1	hypothetical protein
1621554	1640004	18450	Predicted by at least one method		Cys6-1_01094	1639230	1639535	1	hypothetical protein
1621554	1640004	18450	Predicted by at least one method	xerD_2	Cys6-1_01095	1639576	1640004	1	Tyrosine recombinase XerD
2058525	2078210	19685	Predicted by at least one method		Cys6-1_02054	2058525	2058848	-1	hypothetical protein
2058525	2078210	19685	Predicted by at least one method	bltD	Cys6-1_02053	2058896	2059357	-1	Spermine/spermidine acetyltransferase
2058525	2078210	19685	Predicted by at least one method		Cys6-1_02052	2059556	2060062	-1	hypothetical protein
2058525	2078210	19685	Predicted by at least one method		Cys6-1_02051	2060154	2060486	-1	hypothetical protein
2058525	2078210	19685	Predicted by at least one method		Cys6-1_02050	2060615	2060941	-1	hypothetical protein
2058525	2078210	19685	Predicted by at least one method		Cys6-1_02049	2061513	2062094	-1	hypothetical protein
2058525	2078210	19685	Predicted by at least one method		Cys6-1_02048	2062142	2062678	-1	hypothetical protein
2058525	2078210	19685	Predicted by at least one method		Cys6-1_02047	2062708	2063601	-1	hypothetical protein
2058525	2078210	19685	Predicted by at least one method		Cys6-1_02046	2063613	2064455	-1	hypothetical protein
2058525	2078210	19685	Predicted by at least one method		Cys6-1_02045	2064464	2064790	-1	hypothetical protein
2058525	2078210	19685	Predicted by at least one method		Cys6-1_02044	2064777	2064968	-1	hypothetical protein
2058525	2078210	19685	Predicted by at least one method		Cys6-1_02043	2064952	2065215	-1	hypothetical protein
2058525	2078210	19685	Predicted by at least one method		Cys6-1_02042	2065212	2065454	-1	hypothetical protein
2058525	2078210	19685	Predicted by at least one method		Cys6-1_02041	2065486	2065764	-1	hypothetical protein

2058525	2078210	19685	Predicted by at least one method		Cys6-1_02040	2065767	2065991	-1	hypothetical protein
2058525	2078210	19685	Predicted by at least one method		Cys6-1_02039	2065988	2066506	-1	hypothetical protein
2058525	2078210	19685	Predicted by at least one method		Cys6-1_02038	2066516	2066683	-1	hypothetical protein
2058525	2078210	19685	Predicted by at least one method		Cys6-1_02037	2066747	2067451	-1	hypothetical protein
2058525	2078210	19685	Predicted by at least one method		Cys6-1_02036	2067627	2067821	-1	hypothetical protein
2058525	2078210	19685	Predicted by at least one method		Cys6-1_02035	2067972	2068568	1	hypothetical protein
2058525	2078210	19685	Predicted by at least one method		Cys6-1_02034	2068562	2068795	-1	hypothetical protein
2058525	2078210	19685	Predicted by at least one method		Cys6-1_02033	2069427	2069585	1	hypothetical protein
2058525	2078210	19685	Predicted by at least one method		Cys6-1_02032	2069698	2069928	1	hypothetical protein
2058525	2078210	19685	Predicted by at least one method		Cys6-1_02031	2070140	2070559	1	hypothetical protein
2058525	2078210	19685	Predicted by at least one method		Cys6-1_02030	2070730	2071164	1	hypothetical protein
2058525	2078210	19685	Predicted by at least one method		Cys6-1_02029	2071259	2071462	1	hypothetical protein
2058525	2078210	19685	Predicted by at least one method		Cys6-1_02028	2071585	2071986	1	hypothetical protein
2058525	2078210	19685	Predicted by at least one method		Cys6-1_02027	2072200	2072433	1	hypothetical protein
2058525	2078210	19685	Predicted by at least one method		Cys6-1_02026	2072512	2073630	1	hypothetical protein
2058525	2078210	19685	Predicted by at least one method		Cys6-1_02025	2073712	2073918	1	hypothetical protein
2058525	2078210	19685	Predicted by at least one method		Cys6-1_02024	2074236	2074589	1	hypothetical protein
2058525	2078210	19685	Predicted by at least one method		Cys6-1_02023	2075389	2075796	1	hypothetical protein

2058525	2078210	19685	Predicted by at least one method		Cys6-1_02022	2075896	2076414	1	hypothetical protein
2058525	2078210	19685	Predicted by at least one method		Cys6-1_02021	2076459	2076764	1	hypothetical protein
2058525	2078210	19685	Predicted by at least one method	xerD_3	Cys6-1_02020	2077029	2078210	1	Tyrosine recombinase XerD
591735	643354	51619	IslandPath-DIMOB		Cys6-1_00136	591735	592766	1	hypothetical protein
591735	643354	51619	IslandPath-DIMOB	icaA	Cys6-1_00137	592771	593991	1	Poly-beta-1,6-N-acetyl-D-glucosamine synthase
591735	643354	51619	IslandPath-DIMOB		Cys6-1_00138	593994	594254	1	hypothetical protein
591735	643354	51619	IslandPath-DIMOB	icaB	Cys6-1_00139	594264	595157	1	Poly-beta-1,6-N-acetyl-D-glucosamine N-deacetylase
591735	643354	51619	IslandPath-DIMOB		Cys6-1_00140	595184	596638	1	hypothetical protein
591735	643354	51619	IslandPath-DIMOB	brnQ	Cys6-1_00141	596716	598086	-1	Branched-chain amino acid transport system 2 carrier protein
591735	643354	51619	IslandPath-DIMOB	yjdJ	Cys6-1_00142	598323	598595	-1	putative protein YjdJ
591735	643354	51619	IslandPath-DIMOB		Cys6-1_00143	598595	598867	-1	hypothetical protein
591735	643354	51619	IslandPath-DIMOB	nisZ	Cys6-1_00144	600280	600453	1	Lantibiotic nisin-Z
591735	643354	51619	IslandPath-DIMOB	nisB	Cys6-1_00145	600561	603542	1	Nisin biosynthesis protein NisB
591735	643354	51619	IslandPath-DIMOB	msbA	Cys6-1_00146	603553	605355	1	Lipid A export ATP-binding/permease protein MsbA
591735	643354	51619	IslandPath-DIMOB	nisC	Cys6-1_00147	605348	606592	1	Nisin biosynthesis protein NisC
591735	643354	51619	IslandPath-DIMOB	nisI	Cys6-1_00148	606589	607326	1	Nisin immunity protein
591735	643354	51619	IslandPath-DIMOB	nisP	Cys6-1_00149	607328	609376	1	Nisin leader peptide-processing serine protease NisP
591735	643354	51619	IslandPath-DIMOB	arlR_1	Cys6-1_00150	609445	610131	1	Response regulator ArlR

591735	643354	51619	IslandPath-DIMOB	sasA_1	Cys6-1_00151	610124	611467	1	Adaptive-response sensory-kinase SasA
591735	643354	51619	IslandPath-DIMOB	yxlF	Cys6-1_00152	611566	612243	1	putative ABC transporter ATP-binding protein YxlF
591735	643354	51619	IslandPath-DIMOB		Cys6-1_00153	612245	612973	1	hypothetical protein
591735	643354	51619	IslandPath-DIMOB		Cys6-1_00154	612960	613604	1	hypothetical protein
591735	643354	51619	IslandPath-DIMOB		Cys6-1_00155	614738	615448	1	hypothetical protein
591735	643354	51619	IslandPath-DIMOB		Cys6-1_00156	615493	616548	1	hypothetical protein
591735	643354	51619	IslandPath-DIMOB	bcsA	Cys6-1_00157	616603	618582	1	Cellulose synthase catalytic subunit [UDP-forming]
591735	643354	51619	IslandPath-DIMOB	degA_1	Cys6-1_00158	618679	619635	-1	HTH-type transcriptional regulator DegA
591735	643354	51619	IslandPath-DIMOB	scrB	Cys6-1_00159	619632	621050	-1	Sucrose-6-phosphate hydrolase
591735	643354	51619	IslandPath-DIMOB		Cys6-1_00160	621228	623180	1	hypothetical protein
591735	643354	51619	IslandPath-DIMOB	gmuE_1	Cys6-1_00161	623193	624065	1	Putative fructokinase
591735	643354	51619	IslandPath-DIMOB	hsrA_1	Cys6-1_00162	624527	625894	-1	putative transport protein HsrA
591735	643354	51619	IslandPath-DIMOB	ybbH_1	Cys6-1_00163	625942	626763	-1	putative HTH-type transcriptional regulator YbbH
591735	643354	51619	IslandPath-DIMOB	rbsK/rbiA	Cys6-1_00164	627100	627627	1	Bifunctional ribokinase/ribose-5-phosphate isomerase A
591735	643354	51619	IslandPath-DIMOB	iolU_1	Cys6-1_00165	627600	628577	1	scyllo-inositol 2-dehydrogenase (NADP(+)) IolU
591735	643354	51619	IslandPath-DIMOB		Cys6-1_00166	628823	629308	-1	hypothetical protein
591735	643354	51619	IslandPath-DIMOB		Cys6-1_00167	629667	630503	1	hypothetical protein
591735	643354	51619	IslandPath-DIMOB		Cys6-1_00168	631207	631494	1	hypothetical protein

591735	643354	51619	IslandPath-DIMOB	hin	Cys6-1_00169	631753	632307	-1	DNA-invertase hin
591735	643354	51619	IslandPath-DIMOB		Cys6-1_00170	632569	633021	-1	hypothetical protein
591735	643354	51619	IslandPath-DIMOB		Cys6-1_00171	633484	634089	-1	hypothetical protein
591735	643354	51619	IslandPath-DIMOB		Cys6-1_00172	634465	634656	-1	hypothetical protein
591735	643354	51619	IslandPath-DIMOB	rhaS_1	Cys6-1_00173	635546	636601	-1	HTH-type transcriptional activator RhaS
591735	643354	51619	IslandPath-DIMOB	xylP	Cys6-1_00174	636752	638128	1	Isoprimeverose transporter
591735	643354	51619	IslandPath-DIMOB		Cys6-1_00175	638125	639375	1	hypothetical protein
591735	643354	51619	IslandPath-DIMOB		Cys6-1_00176	639392	640969	1	hypothetical protein
591735	643354	51619	IslandPath-DIMOB	bglK_1	Cys6-1_00177	640970	641875	1	Beta-glucoside kinase
591735	643354	51619	IslandPath-DIMOB	bglA_1	Cys6-1_00178	641897	643354	1	6-phospho-beta-glucosidase BglA
1201794	1211161	9367	IslandPath-DIMOB	topA	Cys6-1_00700	1201794	1203926	1	DNA topoisomerase 1
1201794	1211161	9367	IslandPath-DIMOB	trmFO	Cys6-1_00701	1203998	1205341	1	Methylenetetrahydrofolate--tRNA-(uracil-5-)-methyltransferase TrmFO
1201794	1211161	9367	IslandPath-DIMOB	xerS_1	Cys6-1_00702	1205386	1206456	-1	Tyrosine recombinase XerS
1201794	1211161	9367	IslandPath-DIMOB		Cys6-1_00703	1206937	1207224	-1	hypothetical protein
1201794	1211161	9367	IslandPath-DIMOB	xerS_2	Cys6-1_00704	1207307	1208362	-1	Tyrosine recombinase XerS
1201794	1211161	9367	IslandPath-DIMOB		Cys6-1_00705	1209192	1209587	-1	hypothetical protein
1201794	1211161	9367	IslandPath-DIMOB		Cys6-1_00706	1209825	1210631	-1	hypothetical protein
1201794	1211161	9367	IslandPath-DIMOB		Cys6-1_00707	1210679	1211161	-1	hypothetical protein

1201794	1211161	9367	IslandPath-DIMOB		Cys6-1_00708	1211151	1212827	-1	hypothetical protein
1227591	1249990	22399	IslandPath-DIMOB		Cys6-1_00718	1227591	1228766	-1	hypothetical protein
1227591	1249990	22399	IslandPath-DIMOB		Cys6-1_00719	1228744	1230033	-1	hypothetical protein
1227591	1249990	22399	IslandPath-DIMOB		Cys6-1_00720	1230092	1231324	-1	hypothetical protein
1227591	1249990	22399	IslandPath-DIMOB		Cys6-1_00721	1231294	1231704	-1	hypothetical protein
1227591	1249990	22399	IslandPath-DIMOB	purK_1	Cys6-1_00722	1231724	1233001	-1	N5-carboxyaminoimidazole ribonucleotide synthase
1227591	1249990	22399	IslandPath-DIMOB	ldcA	Cys6-1_00723	1233123	1234070	-1	Murein tetrapeptide carboxypeptidase
1227591	1249990	22399	IslandPath-DIMOB	asnS_1	Cys6-1_00724	1234089	1235087	-1	Asparagine--tRNA ligase
1227591	1249990	22399	IslandPath-DIMOB	ubiE_2	Cys6-1_00725	1235122	1235937	-1	Ubiquinone/menaquinone biosynthesis C-methyltransferase UbiE
1227591	1249990	22399	IslandPath-DIMOB		Cys6-1_00726	1236371	1237285	1	hypothetical protein
1227591	1249990	22399	IslandPath-DIMOB		Cys6-1_00727	1237441	1237962	-1	hypothetical protein
1227591	1249990	22399	IslandPath-DIMOB		Cys6-1_00728	1237949	1238812	-1	hypothetical protein
1227591	1249990	22399	IslandPath-DIMOB	ltrA	Cys6-1_00729	1239244	1241043	-1	Group II intron-encoded protein LtrA
1227591	1249990	22399	IslandPath-DIMOB		Cys6-1_00730	1241581	1241979	-1	hypothetical protein
1227591	1249990	22399	IslandPath-DIMOB		Cys6-1_00731	1242029	1242361	-1	hypothetical protein
1227591	1249990	22399	IslandPath-DIMOB		Cys6-1_00732	1242539	1242628	-1	hypothetical protein
1227591	1249990	22399	IslandPath-DIMOB		Cys6-1_00733	1242659	1242979	-1	hypothetical protein
1227591	1249990	22399	IslandPath-DIMOB		Cys6-1_00734	1243368	1244282	-1	hypothetical protein

1227591	1249990	22399	IslandPath-DIMOB	hchA	Cys6-1_00735	1244283	1244960	-1	Molecular chaperone Hsp31 and glyoxalase 3
1227591	1249990	22399	IslandPath-DIMOB		Cys6-1_00736	1244957	1245274	-1	hypothetical protein
1227591	1249990	22399	IslandPath-DIMOB	ceo	Cys6-1_00737	1245749	1246678	-1	N(5)-(carboxyethyl)ornithine synthase
1227591	1249990	22399	IslandPath-DIMOB		Cys6-1_00738	1247055	1247606	-1	Stress response regulator gls24
1227591	1249990	22399	IslandPath-DIMOB		Cys6-1_00739	1247627	1247815	-1	hypothetical protein
1227591	1249990	22399	IslandPath-DIMOB		Cys6-1_00740	1247825	1248385	-1	hypothetical protein
1227591	1249990	22399	IslandPath-DIMOB		Cys6-1_00741	1248415	1248654	-1	hypothetical protein
1227591	1249990	22399	IslandPath-DIMOB	dkgB	Cys6-1_00742	1249148	1249990	1	2,5-diketo-D-gluconic acid reductase B
1621554	1640004	18450	IslandPath-DIMOB	arlR_2	Cys6-1_01063	1621554	1622246	-1	Response regulator ArlR
1621554	1640004	18450	IslandPath-DIMOB		Cys6-1_01064	1622402	1622932	-1	hypothetical protein
1621554	1640004	18450	IslandPath-DIMOB	rpmE2	Cys6-1_01065	1623063	1623308	-1	50S ribosomal protein L31 type B
1621554	1640004	18450	IslandPath-DIMOB		Cys6-1_01066	1623464	1623928	-1	hypothetical protein
1621554	1640004	18450	IslandPath-DIMOB		Cys6-1_01067	1624010	1624372	-1	hypothetical protein
1621554	1640004	18450	IslandPath-DIMOB		Cys6-1_01068	1624857	1625387	-1	hypothetical protein
1621554	1640004	18450	IslandPath-DIMOB	ypbG	Cys6-1_01069	1625448	1626284	-1	putative protein YpbG
1621554	1640004	18450	IslandPath-DIMOB		Cys6-1_01070	1626935	1627330	1	hypothetical protein
1621554	1640004	18450	IslandPath-DIMOB		Cys6-1_01071	1627999	1628268	-1	hypothetical protein
1621554	1640004	18450	IslandPath-DIMOB		Cys6-1_01072	1628589	1628837	1	hypothetical protein

1621554	1640004	18450	IslandPath-DIMOB		Cys6-1_01073	1629847	1630218	1		hypothetical protein
1621554	1640004	18450	IslandPath-DIMOB		Cys6-1_01074	1630468	1630653	-1		hypothetical protein
1621554	1640004	18450	IslandPath-DIMOB		Cys6-1_01076	1630891	1631169	-1		hypothetical protein
1621554	1640004	18450	IslandPath-DIMOB		Cys6-1_01080	1632233	1633018	-1		hypothetical protein
1621554	1640004	18450	IslandPath-DIMOB		Cys6-1_01081	1633075	1633293	-1		hypothetical protein
1621554	1640004	18450	IslandPath-DIMOB		Cys6-1_01082	1633306	1633527	-1		hypothetical protein
1621554	1640004	18450	IslandPath-DIMOB		Cys6-1_01083	1633543	1634292	-1		hypothetical protein
1621554	1640004	18450	IslandPath-DIMOB		Cys6-1_01084	1634395	1635126	-1		hypothetical protein
1621554	1640004	18450	IslandPath-DIMOB		Cys6-1_01085	1635605	1635844	-1		hypothetical protein
1621554	1640004	18450	IslandPath-DIMOB		Cys6-1_01086	1635962	1636231	1		hypothetical protein
1621554	1640004	18450	IslandPath-DIMOB		Cys6-1_01087	1636352	1636507	1		hypothetical protein
1621554	1640004	18450	IslandPath-DIMOB		Cys6-1_01088	1636727	1637113	1		hypothetical protein
1621554	1640004	18450	IslandPath-DIMOB		Cys6-1_01089	1637290	1637853	1		hypothetical protein
1621554	1640004	18450	IslandPath-DIMOB		Cys6-1_01090	1637883	1637981	-1		hypothetical protein
1621554	1640004	18450	IslandPath-DIMOB		Cys6-1_01091	1637990	1638232	-1		hypothetical protein
1621554	1640004	18450	IslandPath-DIMOB		Cys6-1_01092	1638204	1638407	-1		hypothetical protein
1621554	1640004	18450	IslandPath-DIMOB		Cys6-1_01093	1638870	1639016	1		hypothetical protein
1621554	1640004	18450	IslandPath-DIMOB		Cys6-1_01094	1639230	1639535	1		hypothetical protein

1621554	1640004	18450	IslandPath-DIMOB	xerD_2	Cys6-1_01095	1639576	1640004	1	Tyrosine recombinase XerD
2058525	2078210	19685	IslandPath-DIMOB		Cys6-1_02054	2058525	2058848	-1	hypothetical protein
2058525	2078210	19685	IslandPath-DIMOB	bltD	Cys6-1_02053	2058896	2059357	-1	Spermine/spermidine acetyltransferase
2058525	2078210	19685	IslandPath-DIMOB		Cys6-1_02052	2059556	2060062	-1	hypothetical protein
2058525	2078210	19685	IslandPath-DIMOB		Cys6-1_02051	2060154	2060486	-1	hypothetical protein
2058525	2078210	19685	IslandPath-DIMOB		Cys6-1_02050	2060615	2060941	-1	hypothetical protein
2058525	2078210	19685	IslandPath-DIMOB		Cys6-1_02049	2061513	2062094	-1	hypothetical protein
2058525	2078210	19685	IslandPath-DIMOB		Cys6-1_02048	2062142	2062678	-1	hypothetical protein
2058525	2078210	19685	IslandPath-DIMOB		Cys6-1_02047	2062708	2063601	-1	hypothetical protein
2058525	2078210	19685	IslandPath-DIMOB		Cys6-1_02046	2063613	2064455	-1	hypothetical protein
2058525	2078210	19685	IslandPath-DIMOB		Cys6-1_02045	2064464	2064790	-1	hypothetical protein
2058525	2078210	19685	IslandPath-DIMOB		Cys6-1_02044	2064777	2064968	-1	hypothetical protein
2058525	2078210	19685	IslandPath-DIMOB		Cys6-1_02043	2064952	2065215	-1	hypothetical protein
2058525	2078210	19685	IslandPath-DIMOB		Cys6-1_02042	2065212	2065454	-1	hypothetical protein
2058525	2078210	19685	IslandPath-DIMOB		Cys6-1_02041	2065486	2065764	-1	hypothetical protein
2058525	2078210	19685	IslandPath-DIMOB		Cys6-1_02040	2065767	2065991	-1	hypothetical protein
2058525	2078210	19685	IslandPath-DIMOB		Cys6-1_02039	2065988	2066506	-1	hypothetical protein
2058525	2078210	19685	IslandPath-DIMOB		Cys6-1_02038	2066516	2066683	-1	hypothetical protein

2058525	2078210	19685	IslandPath-DIMOB		Cys6-1_02037	2066747	2067451	-1	hypothetical protein
2058525	2078210	19685	IslandPath-DIMOB		Cys6-1_02036	2067627	2067821	-1	hypothetical protein
2058525	2078210	19685	IslandPath-DIMOB		Cys6-1_02035	2067972	2068568	1	hypothetical protein
2058525	2078210	19685	IslandPath-DIMOB		Cys6-1_02034	2068562	2068795	-1	hypothetical protein
2058525	2078210	19685	IslandPath-DIMOB		Cys6-1_02033	2069427	2069585	1	hypothetical protein
2058525	2078210	19685	IslandPath-DIMOB		Cys6-1_02032	2069698	2069928	1	hypothetical protein
2058525	2078210	19685	IslandPath-DIMOB		Cys6-1_02031	2070140	2070559	1	hypothetical protein
2058525	2078210	19685	IslandPath-DIMOB		Cys6-1_02030	2070730	2071164	1	hypothetical protein
2058525	2078210	19685	IslandPath-DIMOB		Cys6-1_02029	2071259	2071462	1	hypothetical protein
2058525	2078210	19685	IslandPath-DIMOB		Cys6-1_02028	2071585	2071986	1	hypothetical protein
2058525	2078210	19685	IslandPath-DIMOB		Cys6-1_02027	2072200	2072433	1	hypothetical protein
2058525	2078210	19685	IslandPath-DIMOB		Cys6-1_02026	2072512	2073630	1	hypothetical protein
2058525	2078210	19685	IslandPath-DIMOB		Cys6-1_02025	2073712	2073918	1	hypothetical protein
2058525	2078210	19685	IslandPath-DIMOB		Cys6-1_02024	2074236	2074589	1	hypothetical protein
2058525	2078210	19685	IslandPath-DIMOB		Cys6-1_02023	2075389	2075796	1	hypothetical protein
2058525	2078210	19685	IslandPath-DIMOB		Cys6-1_02022	2075896	2076414	1	hypothetical protein
2058525	2078210	19685	IslandPath-DIMOB		Cys6-1_02021	2076459	2076764	1	hypothetical protein
2058525	2078210	19685	IslandPath-DIMOB	xerD_3	Cys6-1_02020	2077029	2078210	1	Tyrosine recombinase XerD

1223353	1228766	5413	SIGI-HMM		Cys6-1_00716	1223353	1225290	-1	hypothetical protein
1223353	1228766	5413	SIGI-HMM		Cys6-1_00718	1227591	1228766	-1	hypothetical protein
1223353	1228766	5413	SIGI-HMM		Cys6-1_00719	1228744	1230033	-1	hypothetical protein

Table S4. Antibiotic susceptibility of the *L. lactis* UTNCys6-1 strain

**Antimicrobial E-test strip**	**MIC (mg/L)**	**Susceptibility***	**EFSA cut-off values (mg/L) *Lactococcus* [EFSA, 2012]****
Amoxicillin	0.5	S	4
Amoxicillin: clavulanic acid	0.016	S	4
Ampicillin	0.5	S	2
Cefotaxime	0.002	S	16
Erythromycin	2	S	1
Gentamycin	48	R	32
Penicillin	0.25	S	0.25
Tetracycline	32	S	32

* Susceptible (S): a bacterial strain is defined as susceptible when it is inhibited at a concentration of a specific antimicrobial equal to or lower than the established cut-off value (S ≤ x mg / L). Resistant (R): a bacterial strain is defined as resistant when it is not inhibited at a concentration of a specific antimicrobial above the established cut-off value (R> x mg / L). ** MIC reference for *Lactococcus.*

Table S5. A. Summary of the biosynthetic gene clusters, type and metabolic compounds detected with antiSMASH in the A) UTNCys6-1 genome; B. *L. lactis* ATCC11474 genome.

A.

Sample contig	Reference MiBIG cluster	RiPP-Type	Similarity score	Product	Compound(s)	Organism
contig 1.1	BGC0000554.1	Betalactone	0.16	RiPP	SRO15-3108	*Streptomyces filamentosus NRRL 15998*
	BGC0001579.1		0.16	RiPP	duramycin	*Streptomyces cinnamoneus*
	BGC0000501.1		0.16	RiPP	catenulipeptin	*Catenulispora acidiphila DSM 44928*
	BGC0002049.1		0.16	RiPP	microviridin N9, microviridin N8, microviridin N7, microviridin N6, microviridin N5, microviridin N4, microviridin N3	*Nostoc punctiforme PCC 73102*
	BGC0000617.1		0.15	RiPP	coagulin	*Bacillus coagulans*
	BGC0000558.1		0.15	RiPP	sublancin 168	*Bacillus subtilis subsp. subtilis str. 168*
	BGC0000571.1		0.15	RiPP	burhizin	*Paraburkholderia rhizoxinica HKI 454*
	BGC0001494.1		0.15	RiPP	acinetodin	*Acinetobacter gyllenbergii CIP 110306*
	BGC0001850.1		0.15	Other	diazaquinomycin A, diazaquinomycin E, diazaquinomycin F, diazaquinomycin G	*Streptomyces sp. F001*
	BGC0000548.1		0.14	RiPP	salivaricin A	*Streptococcus salivarius*
contig 1.2	BGC0000535.1	Lanthipeptide-class-I	0.88	RiPP	nisin A	*Lactococcus lactis subsp. lactis*
	BGC0000538.1		0.79	RiPP	nisin Z	*Lactococcus lactis*
	BGC0000536.1		0.63	RiPP	nisin Q	*Lactococcus lactis*
	BGC0001789.1		0.49	RiPP	suicin 90-1330	*Streptococcus suis*
	BGC0000537.1		0.45	RiPP	nisin U	*Streptococcus uberis*
	BGC0000549.1		0.35	RiPP	salivaricin D	*Streptococcus salivarius*
	BGC0001289.1		0.34	RiPP	penisin	*Paenibacillus ehimensis*
	BGC0000544.1		0.3	RiPP	Planosporicin	*Planomonospora alba*
	BGC0000560.1		0.27	RiPP	subtilomycin	*Bacillus subtilis*
	BGC0000528.1		0.25	RiPP	michiganin A	*Clavibacter michiganensis subsp. michiganensis NCPPB 382*
contig 1.3	BGC0000286.1	T3PKS	0.23	Polyketide	viguiepinol	*Streptomyces sp. KO-3988*
	BGC0000764.1		0.2	Saccharide	exopolysaccharide	*Lactococcus lactis*

	BGC0000867.1		0.18	Other	polyhydroxyalkanoic acids	*Ectothiorhodospira shaposhnikovii*
	BGC0000866.1		0.17	Other	polyhydroxyalkanoate	*Burkholderia sp. DSM 9242*
	BGC0001120.1		0.16	NRP, Polyketide	burkholderic acid	*Burkholderia thailandensis E264*
	BGC0001080.1		0.14	Other	endophenazine A, endophenazine B	*Streptomyces anulatus*
	BGC0001076.1		0.14	Other	liposidomycin B, liposidomycin A, liposidomycin C, liposidomycin Z, liposidomycin H, liposidomycin G, liposidomycin L, liposidomycin M, liposidomycin K, liposidomycin N	*Streptomyces sp. SN-1061M*
	BGC0000872.1		0.14	Other	A-90289 A, A-90289 B	*Streptomyces sp. SANK 60405*
	BGC0000934.1		0.14	Other	5-acetyl-5,10-dihydrophenazine-1-carboxylic acid, 5-(2-hydroxyacetyl)-5,10-dihydrophenazine-1-carboxylic acid, endophenazine A1, endophenazine F, endophenazine G	*Kitasatospora sp. HKI 714*
contig 2.1	BGC0001535.1	**RaS-RiPP**	0.23	Other	branched-chain fatty acids	*Streptomyces filamentosus*
	BGC0001534.1		0.19	Other	branched-chain fatty acids	*Streptomyces filamentosus*
	BGC0001229.1		0.17	RiPP	SBI-06990 A1, SBI-06989 A2	*Streptomyces bingchenggensis BCW-1*
	BGC0000897.1		0.17	Other	dehydrophos	*Streptomyces luridus*
	BGC0000497.1		0.17	RiPP	BHT-Aα, BHT-Aβ	*Streptococcus ratti*
	BGC0001356.1		0.16	RiPP	paeninodin	*Paenibacillus dendritiformis C454*
	BGC0000540.1		0.16	RiPP	paenibacillin	*Paenibacillus polymyxa OSY-DF*
	BGC0001889.1		0.16	RiPP	fusilassin	*Thermobifida fusca YX*
	BGC0001645.1		0.16	RiPP	lagmysin	*Streptomyces sp. NRRL S-118*
	BGC0001618.1		0.16	RiPP	hyicin 3682	*Staphylococcus hyicus*
contig 5.1	BGC0001209.1	**RaS-RiPP**	0.28	RiPP	streptide	*Streptococcus thermophilus LMD-9*
	BGC0000902.1		0.17	Other	flaviolin	*Saccharopolyspora erythraea NRRL 2338*
	BGC0000285.1		0.14	Polyketide, Saccharide	flaviolin rhamnoside, 3,3'-diflaviolin, flaviolin	*Saccharopolyspora erythraea*
	BGC0000559.1		0.13	RiPP	subtilin	*Bacillus subtilis subsp. spizizenii ATCC 6633*
	BGC0000938.1		0.11	Other	fosfomycin	*Streptomyces fradiae*
	BGC0000511.1		0.11	RiPP	ericin S	*Bacillus subtilis*

	BGC0000343.1		0.08	NRP	enterobactin	*Pseudomonas sp. J465*
	BGC0001650.1		0.08	NRP	Le-pyrrolopyrazines	*Lysobacter enzymogenes*
	BGC0001895.1		0.08	Polyketide	huanglongmycin A, huanglongmycin B, huanglongmycin C	*Streptomyces sp. CB09001*
	BGC0000949.1		0.07	NRP	A-500359 A, A-500359 B	*Streptomyces griseus*

B.

Sample contig	Reference MiBIG cluster	RiPP-Type	Similarity score	Product	Compound(s)	Organism
Region 1.1	BGC0000286.1	T3PKS	0.23	Polyketide	viguiepinol	*Streptomyces sp. KO-3988*
	BGC0000764.1		0.2	Saccharide	exopolysaccharide	*Lactococcus lactis*
	BGC0000867.1		0.18	Other	polyhydroxyalkanoic acids	*Ectothiorhodospira shaposhnikovii*
	BGC0000866.1		0.17	Other	polyhydroxyalkanoate	*Burkholderia sp. DSM 9242*
	BGC0001863.1		0.15	RiPP, Terpene	bacillicn CER074	*Bacillus mycoides*
	BGC0001080.1		0.14	Other (Phenazine)	endophenazine A, endophenazine B	*Streptomyces anulatus*
	BGC0001104.1		0.14	NRP, Polyketide	myxovirescin A1	*Myxococcus xanthus DK 1622*
	BGC0001076.1		0.14	Other (Nucleoside)	liposidomycin B, liposidomycin A, liposidomycin C, liposidomycin Z, liposidomycin H, liposidomycin G, liposidomycin L, liposidomycin M, liposidomycin K, liposidomycin N	*Streptomyces sp. SN-1061M*
	BGC0000872.1		0.14	Other	A-90289 A, A-90289 B	*Streptomyces sp. SANK 60405*
	BGC0000530.1		0.14	RiPP	mutacin B-Ny266	*Streptococcus mutans N66*
Region 1.2	BGC0001209.1	RaS-RiPP	0.22	RiPP	streptide	*Streptococcus thermophilus LMD-9*
	BGC0000488.1		0.17	RiPP	circularin A	*Clostridium beijerinckii*
	BGC0000554.1		0.16	RiPP	SRO15-3108	*Streptomyces filamentosus NRRL 15998*

	BGC0000616.1		0.16	RiPP	amylocyclicin	*Bacillus velezensis FZB42*
	BGC0000487.1		0.16	RiPP	carnocyclin	*Carnobacterium maltaromaticum*
	BGC0001494.1		0.16	RiPP	acinetodin	*Acinetobacter gyllenbergii CIP 110306*
	BGC0000513.1		0.16	RiPP	Ery-9, Ery-6, Ery-8, Ery-7, Ery-5, Ery-4, Ery-3	*Saccharopolyspora erythraea NRRL 2338*
	BGC0000558.1		0.16	RiPP	sublancin 168	*Bacillus subtilis subsp. subtilis str. 168*
Region 1.3	BGC0000535.1	**Lanthipeptides: putative class I**	1	RiPP	nisin A	*Lactococcus lactis subsp. lactis*
	BGC0000538.1		0.71	RiPP	nisin Z	*Lactococcus lactis*
	BGC0000536.1		0.63	RiPP	nisin Q	*Lactococcus lactis*
	BGC0001789.1		0.49	RiPP	suicin 90-1330	*Streptococcus suis*
	BGC0000537.1		0.46	RiPP	nisin U	*Streptococcus uberis*
	BGC0000549.1		0.35	RiPP	salivaricin D	*Streptococcus salivarius*
	BGC0001289.1		0.34	RiPP	penisin	*Paenibacillus ehimensis*
	BGC0000544.1		0.3	RiPP	planosporicin	*Planomonospora alba*
	BGC0000560.1		0.28	RiPP	subtilomycin	*Bacillus subtilis*
Region 1.4	BGC0001521.1	**Betalactone**	0.17	NRP	auriculamide	*Herpetosiphon aurantiacus DSM 785*
	BGC0000554.1		0.16	RiPP	SRO15-3108	*Streptomyces filamentosus NRRL 15998*
	BGC0001579.1		0.16	RiPP	duramycin	*Streptomyces cinnamoneus*
	BGC0000558.1		0.15	RiPP	sublancin 168	*Bacillus subtilis subsp. subtilis str. 168*
	BGC0000501.1		0.15	RiPP	catenulipeptin	*Catenulispora acidiphila DSM 44928*
	BGC0000617.1		0.15	RiPP	coagulin	*Bacillus coagulans*
	BGC0000571.1		0.15	RiPP	burhizin	*Paraburkholderia rhizoxinica HKI 454*
	BGC0001494.1		0.15	RiPP	acinetodin	*Acinetobacter gyllenbergii CIP 110306*
	BGC0001850.1		0.15	Other (Shikimate-derived)	diazaquinomycin A, diazaquinomycin E, diazaquinomycin F, diazaquinomycin G	*Streptomyces sp. F001*

	BGC0000548.1		0.15	RiPP	salivaricin A	*Streptococcus salivarius*
Region 1.5	BGC0000887.1	RiPP-Type	0.15	Alkaloid	saxitoxin, gonyautoxin 2, gonyautoxin 3, toxin C2, toxin C3, gonyautoxin 5, decarbamoylsaxitoxin, decarbamoylgonyautoxin 2, decarbamoylgonyautoxin 3	*Dolichospermum circinale AWQC131C*
	BGC0000928.1		0.14	Other	T3 toxin	*Cylindrospermopsis raciborskii T3*
	BGC0000188.1		0.14	Alkaloid	saxitoxin, neosaxitoxin, decarbamoylsaxitoxin, gonyautoxin 3, gonyautoxin 2	*Cylindrospermopsis raciborskii T3*
	BGC0000207.1		0.14	Polyketide	chartreusin	*Streptomyces chartreusis*
	BGC0001547.1		0.13	Terpene	citreohybridonol	*Aspergillus stellatus*
	BGC0000631.1		0.08	Terpene	botrydial	*Botrytis cinerea B05.10*
	BGC0000507.1		0.07	RiPP	epicidin	*Staphylococcus epidermidis*
	BGC0001926.1		0.05	Polyketide	chrodrimanin B	*Talaromyces verruculosus*
	BGC0002002.1		0.05	NRP	1,6-phenazinedimethanol, fontizine A	*Serratia fonticola*
	BGC0001591.1		0.05	Other	fatty acid enol ester	*uncultured bacterium CSLC2*
Region 1.6	BGC0001534.1	RaS-RiPP	0.19	Other	branched-chain fatty acids	*Streptomyces filamentosus*
	BGC0001535.1		0.17	Other	branched-chain fatty acids	*Streptomyces filamentosus*
	BGC0000620.1		0.15	RiPP	lacticin Q	*Lactococcus lactis*
	BGC0001628.1		0.1	NRP	JBIR-78, JBIR-95	*Kibdelosporangium sp. AK-AA56*
	BGC0000700.1		0.1	Saccharide	istamycin	*Streptomyces tenjimariensis*
	BGC0000111.1		0.09	Polyketide	neocarzilin A, neocarzilin B	*Streptomyces carzinostaticus*
	BGC0001567.1		0.08	NRP	cysteoamide	*Streptomyces lincolnensis*
	BGC0000185.1		0.08	Polyketide	tartrolon	*Teredinibacter turnerae T7901*
	BGC0001737.1		0.08	NRP, Polyketide	phenalamide	*Corallococcus coralloides*

	BGC0001394.1		0.08	NRP, Polyketide	phenalamide A2	*Myxococcus stipitatus DSM 14675*
Region 1.7	BGC0001209.1	**RaS-RiPP**	0.24	RiPP	streptide	*Streptococcus thermophilus LMD-9*
	BGC0001929.1		0.21	RiPP	WGK	*Streptococcus ferus*
	BGC0000780.1		0.15	Saccharide	O&K-antigen	*Vibrio parahaemolyticus*
	BGC0000779.1		0.15	Saccharide	lipopolysaccharide	*Aeromonas piscicola*
	BGC0000938.1		0.13	Other (Aminocoumarin)	fosfomycin	*Streptomyces fradiae*
	BGC0000343.1		0.07	NRP	enterobactin	*Pseudomonas sp. J465*
	BGC0000982.1		0.06	NRP, Polyketide	cystothiazole A	*Cystobacter fuscus*
	BGC0001055.1		0.05	NRP, Polyketide	yersiniabactin	*Escherichia coli*
	BGC0000806.1		0.05	Saccharide	phosphonoglycans	*Glycomyces sp. NRRL B-16210*

Table S6. Genomes used for pangenome analysis

GenBank (Genome Assembly)	Strain	Isolation source
GCA_016406265.1 (ASM1640626v1)	*Lactococcus lactis strain* WiKim0098	Kimchi
GCA_002148215.1 (ASM214821v1)	*Lactococcus lactis subsp. lactis bv. Diacetylactis* FM03	Cheese
GCA_000468955.1(ASM46895v1)	*Lactococcus lactis* subsp. *cremoris* KW2	Plant
GCA_001456525.1(ASM145652v1)	*Lactococcus lactis* subsp. *lactis* K337	kimchi
GCA_001456385.1 (ASM145638v1)	*Lactococcus lactis* subsp. *lactis* ATCC19437	dairy
GCA_001456615.1 (ASM145661v1)	*Lactococcus lactis* subsp. *lactis* KF282	mustard
GCA_002078975.2 (ASM207897v2)	*Lactococcus lactis* subsp. *lactis* UC06	dairy
GCA_003176835.1(ASM317683v1)	*Lactococcus lactis* subsp. *lactis* 14B4	almonds
GCA_000447905.1(LLDT2.01)	*Lactococcus lactis* subsp. *lactis bv. diacetylactis* TIFN2	cheese
GCA_000478255.2(ASM47825v2)	*Lactococcus lactis* subsp. *lactis* NCDO 2118	frozen peas
GCA_015476255.1(ASM1547625v1)	*Lactococcus lactis* subsp. *lactis* L19	intestine fish
